# Challenges and advances in optical 3D mesoscale imaging

**DOI:** 10.1111/jmi.13109

**Published:** 2022-05-05

**Authors:** Sebastian Munck, Christopher Cawthorne, Abril Escamilla‐Ayala, Axelle Kerstens, Sergio Gabarre, Katrina Wesencraft, Eliana Battistella, Rebecca Craig, Emmanuel G. Reynaud, Jim Swoger, Gail McConnell

**Affiliations:** ^1^ VIB‐KU Leuven Center for Brain & Disease Research Light Microscopy Expertise Unit & VIB BioImaging Core Leuven Belgium; ^2^ KU Leuven Department of Neurosciences Leuven Brain Institute Leuven Belgium; ^3^ MoSAIC‐Molecular Small Animal Imaging Centre KU Leuven Leuven Belgium; ^4^ Department of Physics, SUPA University of Strathclyde Glasgow UK; ^5^ School of Biomolecular and Biomedical Science University College Dublin Dublin Belfield Ireland; ^6^ European Molecular Biology Laboratory (EMBL) Barcelona Barcelona Spain

**Keywords:** 3D imaging, absorption, clearing, light sheet microscopy, Mesolens, mesoscale, optical projection tomography, scattering

## Abstract

Optical mesoscale imaging is a rapidly developing field that allows the visualisation of larger samples than is possible with standard light microscopy, and fills a gap between cell and organism resolution. It spans from advanced fluorescence imaging of micrometric cell clusters to centimetre‐size complete organisms. However, with larger volume specimens, new problems arise. Imaging deeper into tissues at high resolution poses challenges ranging from optical distortions to shadowing from opaque structures. This manuscript discusses the latest developments in mesoscale imaging and highlights limitations, namely labelling, clearing, absorption, scattering, and also sample handling. We then focus on approaches that seek to turn mesoscale imaging into a more quantitative technique, analogous to quantitative tomography in medical imaging, highlighting a future role for digital and physical phantoms as well as artificial intelligence.

## Lay description

This review discusses the state of the art of an emerging field called mesoscale imaging. Mesoscale imaging refers to the trend towards imaging ever‐larger samples that exceed the classic microscopy domain and is also referred to as ‘mesoscopic imaging’. In optical imaging, this refers to objects between the microscopic and macroscopic scale that are imaged with subcellular resolution; in practice, this implies the imaging of objects from millimetre up to cm size with μm or nm resolution. As such, the mesoscopy field spans the boundary between classic ‘biological’ imaging and preclinical ‘biomedical’ imaging, typically utilising lower magnification objective lenses with a bigger field of view. We discuss the types of samples currently imaged with examples, and highlight how this type of imaging fills the gap between microscopic and macroscopic imaging, allowing further insight into the organisation of tissues in an organism. We also discuss the challenges of imaging such large samples, from sample handling to labelling and optical phenomena that stand in the way of quantitative imaging. Finally, we put the current state of the art into context within the neighbouring fields and outline future developments, such as the use of ‘phantom’ test samples and artificial intelligence for image analysis that will underpin the quality of mesoscale imaging.

## 1 INTRODUCTION – WHAT IS OPTICAL MESOSCALE IMAGING?

Imaging in life sciences is increasingly turning towards 3D cultures,^1^, ^2^, ^3^ whole organs^4^, ^5^, ^6^, ^7^, ^8^, ^9^, ^10^ and whole animals^11^ as more relevant models to validate findings from 2D cultures and to visualise development at the system level. For example, the development of small animal models (e.g. *Caenorhabditis elegans*) and the determination of spatial gene expression patterns in tissue have revealed gene interactions that determine the life cycles of parasites.^12^ Importantly, the recent types of (model) organisms used are increasingly diverse, and research horizons are expanding to the study of later developmental stages and thus more complex organ structures. All of these pose new challenges for 3D optical imaging.

**FIGURE 1:**
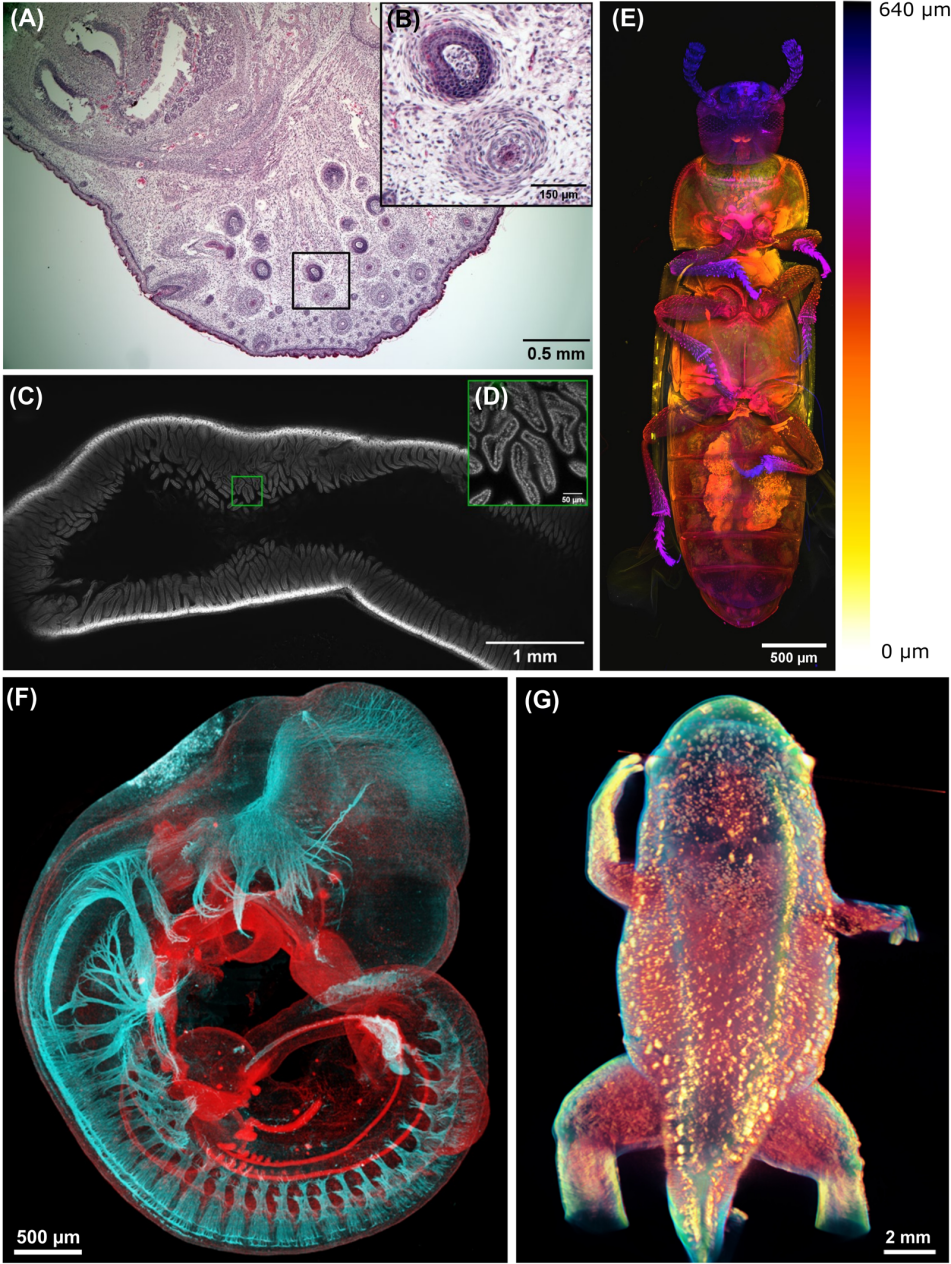
Examples of optical mesoscale imaging using the Mesolens, Light‐Sheet Microscopy and OPT. (A) Brightfield Mesolens image of a section of mouse embryo at full term, stained with haematoxylin and eosin. Scale bar = 0.5 mm. (B) Digital zoom into the snout of the embryo (shown with a black box in A). The individual cell nuclei are revealed at this level of digital zoom, and the individual chromatin granules can be seen in the nuclei. Scale bar = 150 μm. (C) A confocal fluorescence Mesolens image of a whole mount of fixed mouse ileum that has been prepared with the nuclear marker Propidium Iodide and cleared using Murray's Clear. The image shows the mesoscale architecture of the ileum at a depth of 350 μm into the specimen, and a region of interest is shown with a box. Scale bar = 1 mm. (D) Region of interest boxed in C after a software zoom, with the cell nuclei in the crypts now clearly visible. Scale bar = 50 μm. (E) Tribolium castaneum, treated with RNAse and stained with Propidium Iodide and cleared with benzyl alcohol/benzyl benzoate (BABB),^13^ imaged with the Mesolens in confocal mode. This image is composed by maximum intensity projection colour‐coded by depth of 160 optical sections taken with an axial separation of 4 μm, forming a z‐stack 640 μm deep. Scale bar = 500 μm. (F) An antibody labelled E10.5 mouse embryo, cleared with BABB, and imaged with Light‐Sheet Microscopy. Cyan: neurofilament. Red: E‐cadherin. The image is a maximum‐value projection through the 3D data set. Scale bar = 500 μm. (G) Autofluorescence of the skin and transmitted light shape of a *Xenopus tropicalis* frogling imaged by OPT displayed in false colours. Scale bar = 2 mm

This trend towards imaging of ever‐larger samples exceeds the classic microscopy domain and is referred to as ‘mesoscopic imaging’. In optical imaging, this refers to objects between the microscopic and macroscopic scale, while imaging with subcellular resolution; in practice, this implies the imaging of objects from millimetre up to cm size with μm or nm resolution. As such, the mesoscopy field spans the boundary between classic ‘biological’ imaging and preclinical ‘biomedical’ imaging, typically utilising lower magnification objective lenses with a bigger field of view. **Figure** **1** shows examples of mesoscale imaging and highlights the variety of samples, approaches and the resolution scale achieved in mesoscale imaging.

Other specimens used in mesoscale imaging include mouse embryos and intact insects, but also frog (*Xenopus)* tadpoles,^14^ froglings,^15^ zebrafish (*Danio rerio)*,^16^ medaka (*Oryzias latipes*),^17^ insect larvae (*Drosophila melanogaster*)^18^, ^19^ and humans (*Homo sapiens*).^20^, ^21^

The samples used in mesoscale imaging not only differ in size and volume but also in body plan, skeleton structure and composition (e.g. chitin). As such, the sheer size of the samples can pose a challenge for handling and orienting them ideally towards the optical readout and to the staining of specific structures. With increasing sample size, difficulties in imaging originating from the sample have more and more impact on light transmission, for example.

**FIGURE 2:**
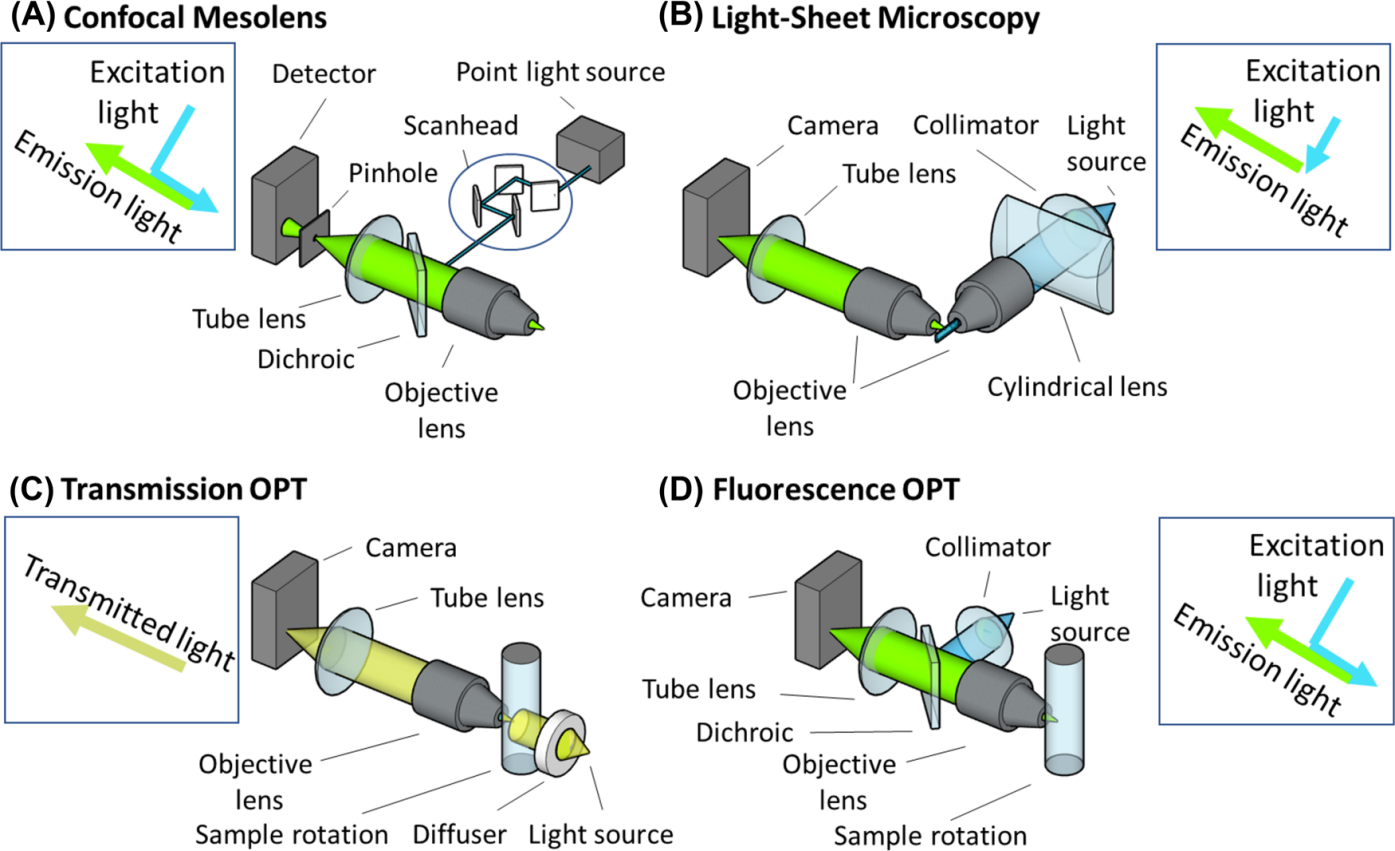
Schematic of optical configurations. (A) Mesolens, a confocal approach using a lens with a unique combination of low magnification and high numerical aperture. (B) Light‐Sheet Microscopy using two lenses with perpendicular orientation, one for illumination and the other for readout. (C, D) Optical Projection Tomography (OPT) approaches. (C) Transmission OPT. (D) Fluorescence OPT. Schematics not to scale, for example, the Mesolens setup is much larger than the other depicted devices

While other techniques such as Computed Tomography (CT), Positron Emission Tomography (PET), Magnetic Resonance Imaging (MRI) and ultrasound‐related technologies can be used to image these kinds of samples, here we focus on optical mesoscale imaging techniques such as the Mesolens, Light‐Sheet Microscopy and Optical Projection Tomography (OPT) strategies (Figures 1 and 2). Additionally, we touch upon the most important challenges for mesoscale imaging and what strategies have already been implemented to overcome such shortcomings. We consider limitations arising from the sample that hamper quantitative imaging, including labelling, absorption, and scattering. We discuss common strategies to tackle absorption and scattering, like intensity correction, clearing, cutting, stripe reduction, multiphoton approaches, adaptive optics strategies and combinations of devices. In addition, we discuss optical mesoscopy in relation to the medical imaging field, specifically imaging preclinical models with microCT, microPET and microSPECT, which have a long tradition of quantitative imaging at the mesoscale range, albeit often at lower spatial resolution than the optical methods highlighted here. We also consider the future use of optical phantoms for calibrating the imaging and analysis strategies for optical mesoscale imaging, including deep learning and other artificial intelligence strategies.^22^

## 2 LABELLING AND LABEL‐FREE OPTICAL MESOSCALE APPROACHES

For a targeted mesoscale imaging approach, one of the problems is labelling structures of interest. Staining is inherently difficult as the tissue forms a diffusion barrier, and penetration of affinity probes may be hampered (a problem that gets worse with increasing sample volume). When comparing affinity probes, small molecules diffuse easier and stain large volumes more readily compared to antibodies, as can be seen for staining of amyloid plaques in brain tissue of mouse models for Alzheimer's disease.^13^, ^23^, ^24^ Consequently, there is some interest in using ‘classic’ immunohistochemistry protocols for mesoscale imaging (Figure 1A–E). Also, staining is typically more difficult for more mature tissue than embryonic samples (Figure 1F) due to the increased stiffness and limited diffusion within them.

Additionally, coelom cavities can be used, for example, for labelling blood vessels through tail vein injections. There is also the possibility of enhancing label infiltration in tissue electrophoretically.^25^ Genetic tagging is another alternative that has become easier through CRISPR strategies. However, despite the progress, sample labelling remains a challenge due to the limited number of established protocols in this emerging field. This is because of the fact that every sample is different and requires adaptations for each species, organ, and developmental stage, thus requiring a divergence from common staining protocols for cell culture.

Consequently, for mesoscale imaging, label‐free imaging presents an opportunity. As such, label‐free imaging can provide complementary information to targeted fluorescent signals. Specifically, by revealing the context in which a fluorescent signal is located, it provides the big picture of the tissue, organ and organism organisation necessary to interpret the targeted fluorescent signals. Examples are the combination of specific fluorescent imaging as in two‐photon imaging with second and third harmonic imaging to access additional structural information.^26^, ^27^, ^28^ Likewise, autofluorescence (Figure 1G) can be used to provide information about the organism in a label‐free manner.

However, label‐free techniques are also increasingly used by themselves to directly access the structure of mesoscale samples. Examples include optical diffraction tomography and quantitative phase imaging approaches used for zebrafish and *C. elegans* imaging.^29^, ^30^ In addition, Optical Coherence Tomography (OCT) can be used to image non‐invasively organs like the eye and the brain, but also increasingly in developmental biology, as for the vascularisation of *Xenopus*.^31^, ^32^

In contrast to current label‐free techniques, the flexibility of the current fluorescence toolbox allows the study of structures with high specificity. For label‐free imaging of complex samples, like whole‐mount preparations, in general, there is a need for benchmarking to pinpoint the (molecular) nature of the signal. However, label‐free imaging can be an interesting option in the context of artificial intelligence (see below).

## 3 APPROACHES FOR OPTICAL MESOSCALE IMAGING

Different approaches have been used to tackle imaging on the mesoscale. In this review, we refer to optical mesoscale imaging techniques and focus primarily on the Mesolens, light‐sheet microscopy, and OPT approaches (Figure 2).

### 3.1 Mesolens – a giant objective lens for 2D and 3D imaging

The Mesolens is a giant, custom‐made objective lens for imaging of tissue volumes up to 100 mm^3^ with subcellular resolution throughout. As such, the Mesolens can be used for most established microscopy techniques, with the advantage of providing a bigger field of view for imaging large specimens with high spatial resolution. Among the established microscopy techniques for 3D imaging, confocal microscopy is currently the technology most used for biological samples. Confocal microscopy can be said to have founded 3D microscopy, with its potential to perform optical sectioning.^33^ The basis of confocal microscopy is the use of a scanned point‐source for illumination and an aperture in the detection path to reject out‐of‐focus light. Consequently, the optical throughput of the microscope and scattering from the tissue determines the amount of light returning from the sample. Therefore, the maximum imaging depth of a confocal microscope has remained around 150–200 μm for most tissue specimens.

Commercial confocal microscopes also have restrictions for the field of view, as the objective lenses used are built to match the resolving capacity of the human eye. As a result, the size of the eye as a detector is used for reference. This has the consequence that multiple tiles or volumes must be stitched together computationally after the acquisition to image larger specimens with an off‐the‐shelf confocal system. Unfortunately, the process of stitching images is not free from problems and artefacts.^34^

Therefore, to image large specimens at high resolution, a complete redesign of the microscope was required (Figure 2A). This led to the development of the Mesolens by McConnell et al.^35^ The Mesolens is an optical lens system that allows for 3D imaging of objects up to 6 mm in diameter, which is comparable to the field of view of a 4× lens and can image samples up to 3 mm thick with a depth resolution of a few microns, instead of the tens of microns currently attained with regular off‐the‐shelf objectives (of comparable magnification) used in confocal microscopes. In addition, the Mesolens has an unusually high numerical aperture (NA = 0.47), allowing subcellular detail to be resolved throughout the entire volume of capture. This means that with the Mesolens, subcellular resolution in 3D for specimens in the mm range is easily achieved. In this sense, the Mesolens is geared towards the challenges of the bigger samples of the future. Applications of the Mesolens to date range from the imaging of whole intact adult *Drosophila melanogaster* ^36^ to transgenic mouse embryos,^35^ imaging of whole mature colony biofilms^37^ and mapping *Tuberculosis sp*. infection in whole lobes of mouse lung.^38^

Moreover, the Mesolens is not restricted to confocal imaging and can be used with other imaging modalities as well. It has been used with a sensor‐shifting CCD camera for widefield, brightfield and epifluorescence imaging of large, but thin specimens. Its high collection efficiency (approximately 20× that of a conventional 4× objective lens) allows imaging of weakly fluorescent specimens with no obvious photobleaching. In addition to confocal imaging, a computational approach to 3D reconstruction with the Mesolens has been demonstrated using the HiLo configuration.^39^ Given the large field of view, establishing a Mesolens‐based light‐sheet is not trivial. Therefore, the HiLo illumination poses a good compromise for transparent specimens with uniform thickness.

A general drawback of the Mesolens is that due to the size of the lens, all optics need to be customised, and respective detection systems are less readily available in the case of use with a camera. In the case of point detection, scan heads and related parts require dedicated solutions; moreover the scanning of large fields of view is slow.

### 3.2 Light‐sheet microscopy

In recent years, the reinvention of light‐sheet microscopy (also known as Selective Plane Illumination Microscopy or, abbreviated, SPIM) has proven to be a very successful way to optically slice thicker samples (beyond the range of a standard confocal microscope) without physically sectioning them for 3D imaging. In light‐sheet microscopy, the sample is illuminated perpendicularly to the imaging axis, decoupling the illumination from the acquisition process.^40^ For this purpose, two perpendicular objective lenses are typically used (Figure 2B). One objective lens is used for illuminating the sample with a sheet of light either generated by a cylindrical lens or by fast lateral scanning of a micrometre‐thin laser beam.^41^, ^42^ The other objective lens is then used to capture the excited fluorescence. Consequently, the light is efficiently used for excitation, and the optical section directly imaged has only limited out‐of‐focus information.

Light‐sheet microscopy is famous for imaging early embryonic development of *Drosophila melanogaster*,^17^, ^43^ and zebrafish,^41^ but also cleared organs like brain tissue.^13^ These applications have triggered an explosion of different implementations and extensions. For example, engineering the thickness of the light‐sheet by using an ‘optical lattice’.^44^ A single‐lens implementation of light‐sheet microscopy called Oblique Plane Microscopy^45^ (OPM) only requires one objective lens near the sample (instead of one each for illumination and detection), allowing more conventional, planar sample mounting. Examples of OPM are Swept Confocally‐Aligned Planar Excitation (SCAPE) microscopy, which was designed for high‐speed volumetric imaging of freely moving and behaving organisms^46^ like tardigrades, and diffractive oblique plane microscopy,^47^ which is an OPM adopted for large samples.

#### 3.2.1 MesoSPIM and related approaches

Different light‐sheet devices have been developed to tackle larger mesoscale samples, including the ultramicroscope from the Dodt group^13^ and the tiling light‐sheet from the Gao group.^48^, ^49^ Likewise, a few commercial setups have been developed to tackle large samples, including commercialisation of the aforementioned approaches from LaVision and 3i, as well as combined approaches using light‐sheet and OPT approaches from Planelight.^50^ One of the recent light‐sheet‐based open hardware developments for large samples is the mesoSPIM (mesoscale Selective Plane Illumination Microscopy) initiative. It optimises the optical sectioning for large samples by synchronising the camera's rolling shutter with the thinnest part of the light‐sheet (its waist), creating an optimally thin light‐sheet at the imaging plane, which would otherwise be impossible to achieve.^51^ As the mesoSPIM mostly images optically cleared specimens, it uses air lenses to allow the use of all kinds of clearing agents as compared to the more commonly used water‐dipping lenses. The disadvantage is that air lenses limit the achievable resolution because of refractive index mismatches and the lower numerical aperture.

Limitations for mesoSPIM, and light‐sheet microscopy in general, are that with larger samples, these systems have less spatial resolution as the working distance of the applicable objective lenses and the resolution are inversely related.^31^, ^52^ A general problem with light‐sheet imaging is the frequent uneven illumination. In addition, the scattering introduced by the sample in a light‐sheet microscope can lead to a progressively defocused light‐sheet with imaging depth, compromising the optical sectioning capability of the setup.

It is an irony that the optical concept of illuminating with a sheet of light perpendicular to the readout axis was originally developed in the early 20th century for imaging nanometre‐size objects by light scattering (using an ‘extreme darkfield’‐style setup), while the reinvented light‐sheet microscope using fluorescence as a readout is hampered by scattering.^53^

One of the advantages of the light‐sheet approach is the speed of imaging that can be achieved.^54^ In general, we believe that many inventions from microscopic light‐sheet imaging^55^ will be reimplemented on the mesoscale.

### 3.3 Optical projection tomography

For imaging of small animals and structures, several techniques from medical imaging have been directly adapted. Micro‐Computed Tomography (microCT) scanning is the longest established of these and uses X‐rays to produce a tomographic map of object density, with a range of available contrast agents to better visualise structure both ex vivo and in vivo.^56^, ^57^ Inspired by the tomographic approach of CT, in 2002 Sharpe and coworkers developed a new technique called OPT.^58^ OPT is a 3D imaging technique utilising a series of 2D views to reconstruct a 3D voxel image computationally. For small samples, images are acquired by rotating the sample, and typically, views are collected from 360°. Images are then reconstructed using the same procedures from the CT world, that is, filtered backprojection and helical rotation algorithms.^59^ The technology can be used in transmitted light (Figure 2C) or fluorescent mode (Figure 2D).

Given that multiple images need to be acquired from different angles, the photostability of the used fluorophores towards bleaching is important. Next to the established use of fluorescent labelling, autofluorescence is used regularly to reveal the structure of the biological specimen. To accommodate out‐of‐focus regions and aberrations, several improvements and plenoptic imaging have been integrated into OPT.^50^, ^60^, ^61^ Regardless, this mode of imaging gives the possibility for quantitative 3D analysis of small model organisms or embryos in their original geometry.^62^ In addition, the reconstructed volumes have a nearly isotropic resolution, which means that the resolution in the *z*‐direction can exceed that of a confocal microscope with similar optics.

One general drawback of OPT is the inherent need for reconstruction. This process can be computationally demanding and uncouples the acquisition process from the 3D visualisation, standing in the way of quickly assessing the quality of the acquisition process.

Another drawback is that the lateral resolution is limited due to the use of telecentric long working distance lenses. To overcome this shortcoming, light‐sheet and OPT have been combined in one device,^63^ the OPTiSPIM. In this way, one can take advantage of the lateral resolution of SPIM and the (isotropic) axial resolution and nonfluorescent contrast imaging of the OPT.

### 3.4 Other related technologies

Mesoscopic imaging is notably an active research field, and multiple approaches are being developed for depicting samples in the mesoscopic size range. Other interesting approaches are Optical Coherence Microscopy (OCM) and photoacoustics, to name two. OCM is poised to provide additional information with images beyond the technical limitations of fluorescence imaging. Implemented in a confocal scanning microscope and using a high NA objective lens, OCM is an interference method that provides 3D reconstructions based on intrinsic contrasting of backscattered coherent light^31^, ^64^ typically in the near‐infrared range. It has been used, for example, to image early embryonic development in a label‐free manner.^64^

Another possibility is to leverage the deep‐reaching capability of ultrasound in combination with high‐resolution imaging. Combining microscopy with optoacoustics might bring together the best of both worlds, allowing deeper penetration and a way to image small animals as a whole.^65^ This technology also holds potential for medical applications.

## 4 OPTICAL MESOSCOPY IMAGING CHALLENGES

Light interacts with matter in multiple ways. We utilise these effects, for example, in the form of fluorescence, when labelling our samples. However, other optical phenomena play a role in mesoscale imaging beyond the desired effects. Here we focus on absorption and scattering as these lead to well‐known artefacts in microscopy, which are even amplified in mesoscale imaging, such as shadowing stemming from bone, chitin and blood vessels and loss of optical performance in the case of scattering.

### 4.1 Absorption and scattering

**FIGURE 3:**
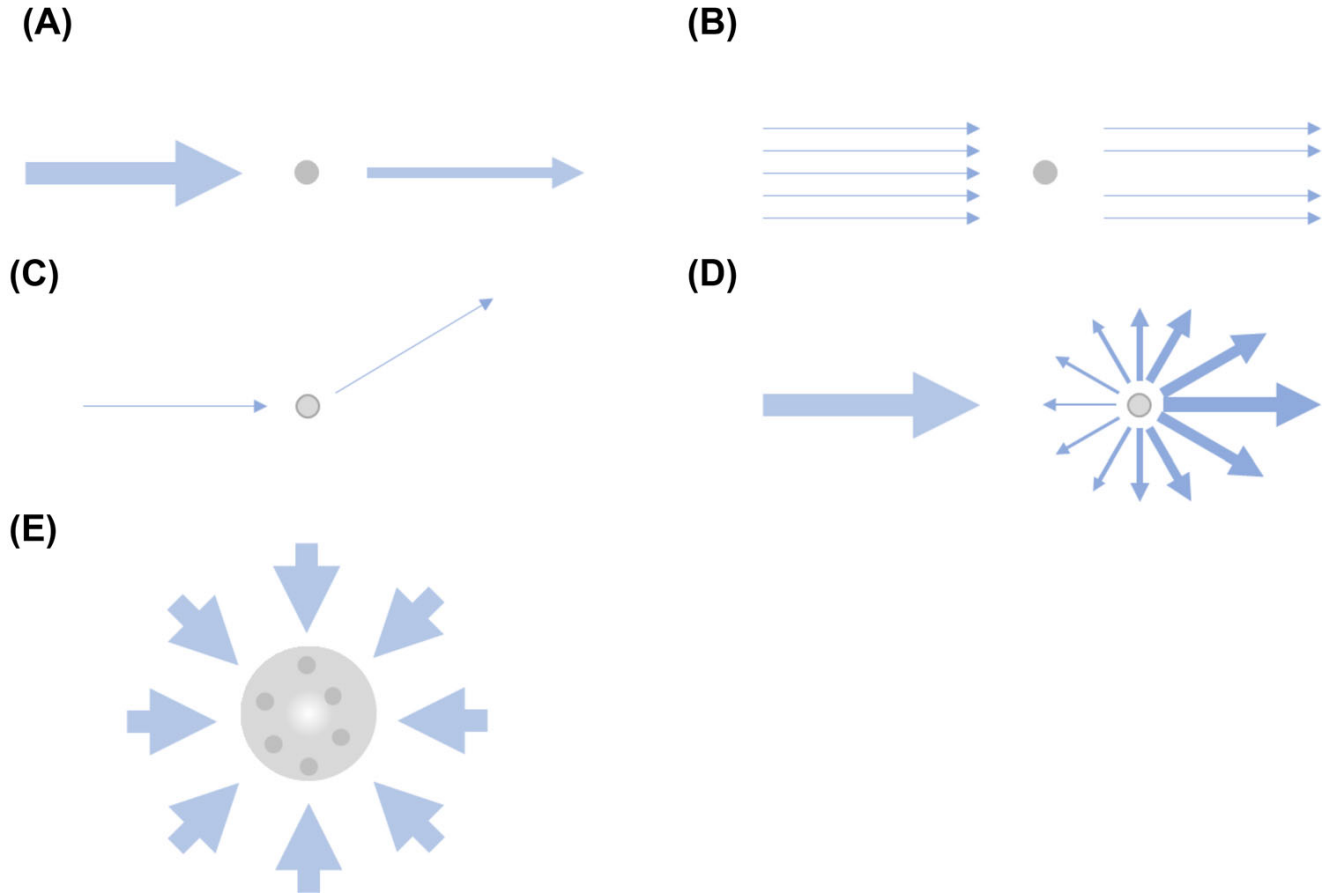
Schematic for absorption and scattering. (A) Absorption. The intensity of incident light is reduced by absorption. (B) Shadowing. For a number of incident photons shadowing is induced by complete absorption by the particle as indicated by the arrows. (C) Scattering. The direction of incident light is changed by a scattering angle by interaction with a particle indicated by the arrows. (D) Depending on the size, shape and density of the particles the light will be scattered differently. Here a Mie type of scattering is depicted, where more forward scattering is happening, indicated by the thickness of the arrows. (E) Sample complexity can lead to unrecoverable regions marked here by the white space in the middle, where absorption and/or scattering block the region from all possible imaging directions

Absorption and scattering of light by mesoscale samples are a central problem for all light‐based mesoscale imaging techniques (Figure 3). The two are often combined and regarded as attenuation or extinction.

Attenuation is the gradual loss of flux through a medium that can be caused by absorption, reflectance, and scattering. In the case of the simple question of light going through a uniform medium, Lambert‐Beer's law quantifies the amount of absorbance A as,

$$A=\varepsilon lC=\log_{10}\frac{I_{0}}{I},$$

with *l* being the optical path length, *ε* being the molar attenuation coefficient for a given wavelength and *C* being the concentration. Further, *I*_0_ being the incident light intensity and *I* being the transmitted light (Figure 3A). However, absorption and scattering often occur together and, in practice, they are regarded together, as the energy *E* of extinction for both phenomena is additive:

$$E_{\mathrm{Exti}\mathrm{ncti}\mathrm{on}}=E_{\mathrm{Scat}\mathrm{teri}\mathrm{ng}}+E_{\mathrm{Abso}\mathrm{rpti}\mathrm{on}}.$$

Consequently, the scattering can be accessed in a similar fashion as the absorption (in the simple transmission case), for example, in turbidimetry. However, the absorbance refers to the amount of light absorbed in the sense of energy of photons taken up as opposed to being reflected or refracted (Figure 3B). In contrast, scattering refers to the light deviating from its path upon interaction with particles depending on the size and shape of the particles.

To emphasise the difference between scattering and absorption, and to describe the difference with Lambert–Beer's law above, the process of scattering can be regarded by two functions, *S*_1_ (*θ,φ*) and *S*_2_ (*θ,φ*) for amplitude and phase, with *θ* being the scattering angle and *φ* being the corresponding azimuthal angle^66^ (Figure 3C). Consequently, scattering is often measured at an angle, as in nephelometry. Importantly, the amplitude of a scattered wave is inversely proportional to the distance of detection. This means that scattered photons can interfere within a complex optical system and continue to propagate at a different angle (*S*_1_ & *S*_2_) while absorbed ones are lost (Lambert–Beer).

In samples imaged by light microscopy, mostly elastic scattering (Rayleigh and Mie‐type) affects the light path, with Mie and Rayleigh referring to approximations of solutions to Maxwell's equations on electromagnetism for differently‐sized particles (Figure 3D). The amount of scattering and the contributions from Rayleigh and Mie scattering for different tissue types have been extensively reported by Jacques.^67^

Next to the weakly scattering effects described above, depending on the tissue and the scale at which the tissue is observed, biological tissues can progressively be considered strongly diffusive media, in which multiple scattering events occur. For infrared imaging, where absorption is less relevant, scattering becomes dominant. Diffuse optical imaging and tomography (DOI; DOT) are non‐invasive techniques that utilise light in the near‐infrared spectral region, allowing interrogation of translucent tissue like breast or brain. In this strong scattering imaging regime, a major concern is lateral interpixel crosstalk, which degrades the resolution. Consequently, major efforts have been put into the assessment of photon transport in these tissues to improve reconstructions.^68^, ^69^, ^70^, ^71^, ^72^ Likewise, Monte Carlo simulations are used in PET along with digital phantoms to evaluate reconstruction/quantification protocols,^73^ and generally, different approaches have been made to model scattering in tissue.^74^ It is of note that beyond the optical imaging regime, scattering can be further distinguished as elastic scattering, where there is no change in the energy, or inelastic, where energy is lost (and the intensity is attenuated) in the process of scattering, as in Compton scattering.

However, for the optical mesoscale imaging highlighted here, mostly weak scattering is relevant and the two processes, absorption, and scattering, affect each optical configuration differently (Figure 2). Thus, it is useful to consider them separately (Figure 3A–D). Practically, when scattering affects the excitation light path, it will lead to a less‐defined excitation spot/sheet, and the focal positions will be less bright. If scattering affects the emission path, the imaged position will be more diffuse. In the case of a transmission image, scattering may appear as absorption if the light is scattered out of the detection path, while photons scattered into or within the detection path increase the background, reducing the relative signal strength. With larger samples, scattering‐related problems typically increase.

In fluorescence, if absorption affects the excitation light path, it means that less light will be available to excite the fluorophore. In the case of emission, it means that less light will reach the detector. Depending on the optical configuration, combinations of these effects may occur. For example, in light‐sheet imaging, scattering affects the thickness of the light‐sheet due to broadening the light‐sheet. In addition, resolution is affected due to lateral crosstalk impacting *xy* information. The latter is a problem for all widefield types of detection schemes. If the sample contains pigments (for example, haemoglobin or melanin) and visible light is used, the signal is attenuated due to the interaction of the light with the pigments. These pigments will lead to photons being absorbed, resulting in a loss of signal for imaging (Figure 3E). Table 1 summarises the effect of absorption and weakly scattering tissue on the different imaging modalities.

**TABLE 1:** Simplified effects of absorption and weakly scattering tissue on the illumination/detection light of the methods reviewed here. The effects are different for each technique

	Absorption	Scattering
Confocal Mesolens	Reduced signals	Reduced signals
Light‐sheet microscopy	Shadowing artefacts	Less confocality
Transmission OPT	Depicted as signal	Depicted as signal
Fluorescence OPT	Less signal	Less optical confinement

Diverse measures have been taken to compensate for scattering and absorption in the different optical approaches. However, one of the most universal ways to tackle this is by clearing the sample.

## 5 DEALING WITH ABSORPTION AND SCATTERING

Clearing is a biochemical process that renders tissue, and even complete animals, significantly transparent by immersing the specimen in a clearing agent. This process was first explored by Spalteholz at the beginning of the last century,^75^ but it is now experiencing a renaissance thanks to new and emerging mesoscale imaging technologies.^76^ Clearing mostly reduces scattering by reducing refractive index mismatches in the tissue and between the tissue and the immersion medium, but procedures that include quenching, bleaching, and oxidation of pigments, thus reducing absorption, have also been developed.^77^ Protocols have been optimised for different cellular materials and for specific tissue types, for example, skin and bone,^78^ as well as the development of clearing methods that preserve the fluorescence signal from fluorescent proteins^79^ or that combine clearing with labelling.^80^ In the context of expansion microscopy,^81^ where the sample is embedded in a hydrogel and swells isotropically in size to permit microscopic imaging of nanoscale structures, the sample is also rendered transparent.^82^

One disadvantage for most clearing protocols is that the sample is fixed, with a few notable exceptions of live clearing.^83^ In addition, clearing does not always work perfectly, with the problems of scattering and absorption persisting to some extent. Finally, clearing is a series of chemical processes that remove or modify the sample composition, which may have some effects on the structure to be imaged.

Another sample‐based approach to deal with absorption and scattering is the usage of fluorophores that are redshifted. Due to less scattering and absorption of biological tissue in the longer wavelength range dyes and fluorophores that can be used in the near‐infrared and infrared range are increasingly adopted.^84^, ^85^

### 5.1 Compensating for the lost signal

Next to sample‐based approaches, the most commonly used strategy to compensate for a reduction in signal strength due to absorption and scattering is either through post‐processing or during the acquisition itself, by increasing either the excitation power or the detection amplification when possible. The goal of this compensation is typically to improve the signal‐to‐noise ratio across the sample. In this section, we describe how each of the mesoscale imaging techniques apply compensation.

#### 5.1.1 Compensation for the Mesolens

In the confocal Mesolens approach, the optical configuration for excitation and emission makes use of the same giant objective lens. As with confocal imaging, the pinhole is placed in an optical plane that is conjugated to the focal plane, and that pinhole then rejects out‐of‐focus information. At the same time, it also rejects scattered light, which seemingly does not originate from the point of excitation. This explains why confocal systems have a limited penetration depth in tissue, as both the excited and the emitted light gets scattered, resulting in dimmer images. The intensity can be increased by using higher powers for excitation to compensate for the loss by scattering at the price of additional light exposure of the specimen, thus causing bleaching and phototoxicity in living specimens. However, photons lost on the emission side cannot be recovered, leading to a poorer signal‐to‐noise ratio. Similarly, increasing the photodetector gain to compensate is usually at the expense of the signal‐to‐noise ratio. These rather crude compensation mechanisms fall short because they do not take the sample structure per se into account. This is because the exact 3D distribution of attenuating structures in the sample is generally not known, so quantitative estimates of how much to increase the excitation light power and/or detection gain cannot be made.

#### 5.1.2 Compensation in light‐sheet microscopy

In light‐sheet microscopy, both the illumination light path as well as the detection light path can be affected by scattering and absorption, with penetration of the light‐sheet being a concern for the illumination, and the imaging depth relative to the detection lens affecting the emitted light traveling through the sample. Although much effort has been spent in obtaining a greater degree of penetration for light‐sheet microscopy,^86^, ^87^ to date, this effort has been focused on obtaining a signal via ‘attenuation compensation’ rather than correcting for inhomogeneity in the sample per se. The presence of differentially attenuating/scattering structures in a mesoscale sample casts ‘shadows’, leading to concealed regions in the sample or distortion of the expected signal strength at the detector. As the typical strategies to address these problems are by multiview imaging,^18^, ^88^, ^89^ it is unlikely to be corrected quantitatively. Using different angles for imaging will effectively decrease the thickness of the sample, therefore, limiting the effect of attenuation, but does not address the size and morphology of attenuating biological structures as such. Remarkably, little attention has been given to date to overcome the effect of such attenuation on mesoscale light‐sheet images.

### 5.2 Stripe reduction

A common problem in light‐sheet imaging is stripes originating from shadows created by absorption, for example, by blood vessels. One way to address this is by using pivot scans that vary the angle of the light‐sheet hitting the sample and therefore limiting the shadow to a cone instead of a stripe.^88^ Another strategy is using diffuse light‐sheets for illumination.^90^ While this helps to improve image quality, the problem is just diluted instead of being fully addressed. Multiview imaging of the sample can also reduce stripes to some extent (Figure 3C–E).^88^, ^89^

### 5.3 Multiphoton excitation

One solution to reach deeper into tissue is to use two‐ or three‐photon fluorescence microscopy.^91^, ^92^ The idea behind these techniques is to use ultrashort pulses of infrared light that are less prone to scatter. Additionally, light in the near‐infrared spectral range is less absorbed than light in the visible spectral range in most biological tissue. The multiphoton effect is then used to excite a spatially confined volume whose point spread function depends on the numerical aperture of the objective lens, the wavelength of illumination, the beam quality of the laser, and the scattering and absorbing properties of the tissue. As the nonlinear absorption effect has a significant probability of happening only in the focal spot of the system and depends on the laser's peak intensity, all fluorescence signals emitted by the sample can, in theory, be detected and used for imaging. Two‐photon strategies have been applied to light‐sheet microscopy^93^ and could be applied to the Mesolens and OPT, as well. However, this strategy has its limitations, as creating long, thin sheets of light with two‐photon illumination is not easy. Gaussian optics can create a long sheet of light extending over several millimetres, but it is too thick to give cellular or subcellular resolution over the field of view. It is also necessary to consider the peak intensity that must be spread over the field of view: this is managed easily by the focused scanning spot in a conventional two‐photon microscope, but the peak intensity must be scaled accordingly for widefield or light‐sheet illumination.^94^ Another challenge in multiphoton microscopy is preserving the ultrashort pulse duration at the specimen plane, but this can be managed through pre‐dispersion compensation using gratings or fibre pulse compressors.^95^, ^96^

### 5.4 Cutting and imaging – HREM

One of the limitations of both two‐photon and confocal microscopy is the axial resolution, which is usually on the order of 0.3–1 μm. In addition, in the 1980s, it became clear that optical sectioning works best for high NA lenses. The problem is that typically high NA lenses have a shorter working distance, while longer working distance lenses have a lower NA, resulting in low‐resolution images. Cutting tissue and imaging the slices is one way around that problem, allowing retrieval of images from inside the tissue with a high NA lens while reducing scattering and absorption. The drawback is the computational challenge to register the images. Alternatively, the blockface can be imaged as in High‐Resolution Episcopic Microscopy (HREM) and related technologies.^97^, ^98^, ^99^, ^100^ These systems can be used to image tissue autofluorescence as well as specific signals from expressed fluorescent proteins targeted to a structure of interest in model organisms and can be combined with confocal or two‐photon imaging. Unfortunately, the technique is dedicated to fixed samples, and artefacts can arise from cutting and embedding samples in a stabilising matrix. For thinner slices, the sample needs to be more rigid which can be achieved, for example, by paraffin embedding or freezing. Interestingly, the combination of two‐photon imaging for brain tissue in an HREM fashion poses a valid alternative for imaging with a cellular resolution.^101^

### 5.5 Adaptive optics

Adaptive optics can be used to correct scattering and distortion stemming from refractive index mismatch in the sample. The concept of adaptive optics originates from astronomy, where an artificial guide star in the form of a laser directed at the night sky is used to measure distortions induced by the atmosphere.^102^ In microscopy, a wavefront sensor is often used to detect the distortions. Consequently, an adaptive optics element corrects the light wavefront to obtain a less distorted image. For the correction, a deformable mirror or a liquid crystal array can be used. Adaptive optics are used extensively in high‐resolution microscopy, for example, in the context of brain imaging^103^ as well as in light‐sheet imaging^84^, ^104^ to improve the achievable resolution. Specifically, for long‐term imaging and following the embryonic development of mice over time, adaptive optics have been instrumental by being able to adapt to the growing and changing specimen.^84^ However, the optimisation of the wavefront for best correction is often slow, which can lead to both phototoxicity and photobleaching of the specimen. Adaptive optics can also be applied to OPT and the Mesolens. For the Mesolens, however, the deformable elements would need to be huge to be compatible with the optics of the Mesolens. This problem may be solved by working with the astronomy community, who originally developed adaptive optical elements for long‐range imaging applications.^102^

### 5.6 Correction in OPT

For OPT, different approaches for correcting scattering and absorption have been implemented. In particular, an optical attenuation map generated via transmission can be used to inform the reconstruction of emission images and, thus, account for non‐uniform absorption.^105^ Moreover, in fluorescence OPT, specific illumination schemes using parallelised semi‐confocal line illumination and detection have been developed to discriminate against scattered photons.^106^

### 5.7 OPTiSPIM

For correcting absorption in light‐sheet imaging, the OPTiSPIM^63^ is an interesting approach. The device is a combined OPT and light‐sheet system. The idea is to measure the absorption using the transmitted light OPT. From that, a 3D mask is generated to correct the intensities of the light‐sheet data. This is remarkable given that corrections come from a measurement‐based system, and the combination of techniques is used to create a sample‐based correction of the data. Scattering in the transmitted light OPT is interpreted as attenuation of the signal, so that the correction of the SPIM data will compensate for the loss of signal in this respect. However, scattering will also lead to less crisp images in the OPT. Although there is no way to distinguish between absorption and scattering in this approach, artefacts can be corrected and image quality improved. This approach can be considered in some ways analogous to the corrections used in medical imaging, where a CT image is used for correcting Singlephoton Emission Computerised Tomography (SPECT) or PET images.

## 6 STRATEGIES FROM MEDICAL IMAGING

Given the similarity of correction between the OPTiSPIM and medical imaging approaches, we thought it worthwhile to look closer at how correction is carried out in medical imaging, and the underlying foundations. Next to corrections, the potential for connecting mesoscale and medical imaging for multimodal imaging has been highlighted recently.^107^

Tomography, the generation of cross‐sectional images via the acquisition of projection data of a penetrating wave/particle, is well established in a range of medical imaging techniques that have now found application at the mesoscale. Conceptually, emission tomography is similar to fluorescence microscopy in the sense that images are created from photons that are emitted from the sample. Then, projection data are used to reconstruct the image, which is corrected for both attenuation and scattering. Alternatively, attenuation correction is incorporated into iterative reconstruction techniques to allow the generation of images that reflect the true geometry and activity of the source. Importantly, the resulting quantitative images are not biased due to depth‐dependent effects, that is, the attenuation of high‐energy photons as they pass through the body to the detector or their mispositioning due to scatter.

**FIGURE 4:**
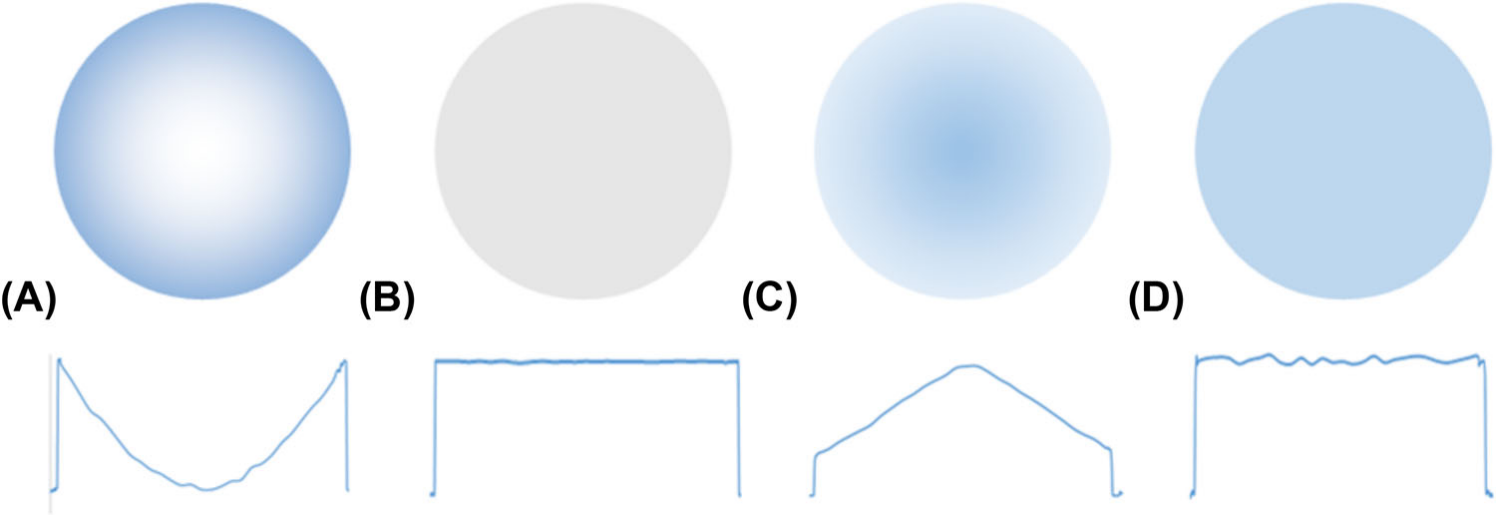
Reconstructed images from a cylinder containing a uniform activity, with corresponding line profiles. (A) Reconstructed PET image without attenuation or correction for scattering. (B) Attenuation map derived from CT image of the same cylinder. (C) Reconstructed PET image corrected for attenuation but uncorrected for scattering effects. (D) Final corrected PET image. Adapted from Ref. (109)

In PET and SPECT, attenuation and scattering are part of the same physical process, that is, the (inelastic) Compton scattering of high‐energy photons as they pass through the body (photoelectric absorption is limited at the photon energies for PET and most SPECT isotopes). Thus, attenuation is a loss of signal as a consequence of the inelastic interaction, while scattering seemingly leads to an increase due to erroneous coincidence detection^108^ (although at energies below 15 keV Rayleigh scattering is dominant in tissue, the angles of deflection are small, and so, in this case, scattered and primary photons are not separated). Without correction, attenuation leads to images where the centre of the object has less apparent activity than the edges, which are overestimated (Figure 4A), and scattering leads to an increase in events towards the centre of the object (Figure 4C).

In PET, correction for attenuation has the largest impact and is made based on an attenuation map of the object, created via a transmission source or by scaling the information from a CT image to the photon energy of the emitter. Due to the unique emission of (almost) coincident photons, attenuation for any line of response can be readily established, and individual projections can then be corrected before image reconstruction (Figure 4C). In SPECT, the situation is more complex due to the lack of these coincident photons. Thus, information from the attenuation map is used as part of the iterative reconstruction of the radioactive image.

Without correction for scattering, uniform density images will demonstrate higher apparent counts towards the centre of the imaged object (Figure 4C). Light scattering is the most difficult phenomenon to correct for in positron and single‐photon emission tomography, involving the incorporation of modelled or measured effects of scattering in an iterative reconstruction.^110^, ^111^ There are two general approaches: the measurement of scattering at a separate energy window(s) set narrowly around the expected peak that can then be scaled and subtracted from the detected peak or modelling approaches involving the calculation of the scattering, for example, based on Klein‐Nishina equations or experimental values. In both cases, the derived terms for the scattering are then incorporated into the image reconstruction. Although attenuation maps created via CT are widely used in preclinical emission tomography, the advent of PET‐Magnetic Resonance Imaging (PET‐MRI) has renewed efforts at correcting for attenuation directly, using only emission data.

OPT, where optically cleared samples are employed so that scattering is reduced, can be considered analogous to transmission (CT) or emission (PET, SPECT) imaging depending on the mode. However, it is useful here to note the differences between the implementation of tomographic approaches in both domains. In (biomedical) emission tomography, quantitative imaging is established as a goal, and corrections, albeit implemented in a range of ways, are considered standard. To this end, clinical (and increasingly preclinical) sites routinely assess the quantitative performance of their instruments using defined standards and phantoms, for example, National Electrical Manufacturers Association (NEMA) NU2, NEMA NU4. As well as allowing comparison between instruments and enabling the standardisation of best practice,^112^ phantoms facilitate the assessment of novel or more complex experimental setups by allowing a robust assessment of any effects on image quality and quantification (for example, multiple animal scanning in preclinical studies).^113^ Consequently, reconstruction algorithms used for OPT are also discussed that include scattering.^106^, ^114^, ^115^

## 7 PERSPECTIVES

### 7.1 Future use of optical phantoms

In medical and preclinical imaging, so‐called imaging phantoms, or just phantoms, are commonly used to evaluate, tune and calibrate the imaging process. Phantoms simulate the imaging properties of human tissue. They are used as standard test objects to validate the performance of imaging devices and often are part of a quality control procedure. Typically, they contain recognisable features that can be used for evaluation^116^ and can mimic a tissue's optical, thermal and mechanical properties.

With phantoms being used as test objects for a specific imaging modality, they need to be tailor‐made for that imaging process. The advent of 3D printing offers new possibilities for microscopy in general^117^ and specifically to create solid or hydrogel tissue phantoms for a multitude of imaging modalities with relative ease. Fabrication allows the optical properties of phantoms to be controlled through the incorporation of scattering and absorbing media. These phantoms and their print files can then easily be shared.

This opportunity has already been picked up in medical imaging, where printed phantoms have been investigated to improve microCT, X‐ray imaging, and OCT,^118^ and similar approaches have been applied for diffuse optical imaging^119^ as well as MRI and CT^120^ and quantitative phase imaging.^121^ The printing process offers an opportunity, specifically, as the resolution of the prints can be in the same range as that of optical mesoscale imaging.

Emerging bioengineering technologies such as 3D bioprinting can create well‐defined 3D tissue structures that can be tailored using a variety of extracellular matrix (ECM) components, including collagen. This enables the design of multilayered tissue phantoms with specific physical characteristics, such as different densities or fibrous content. 3D bioprinted optical tissue phantoms can be printed with fiducial markers deposited within them during the printing process, allowing the assessment of light at different depths and positions within the volume. They can accurately mimic the optical properties of biological tissues for mesoscale imaging device calibration, comparison, validation, and even training for the detection of specific structures. However, 3D bioprinted tissue phantoms are not yet widely adopted in optical imaging.^122^

We believe that phantoms can be used for optical mesoscale imaging to tackle scattering and absorption, similarly to their use in medical imaging. The growing availability, combined with the versatility of 3D bioprinting, can provide multiple approaches for phantoms in optical mesoscale imaging. Consequently, we believe that akin to medical imaging corrections based on phantoms combined with elevated theoretical, and machine learning approaches, or similar strategies could help optical mesoscale imaging become more quantitative. We expect that with more use cases and the ease of creating phantoms, more and more dedicated phantoms will emerge for specific purposes, such as resolution targets, phantoms to calibrate reconstructions and distortions, as well as to allow for registration between modalities.

### 7.2 Analysis and future use of AI

For analysis of mesoscale data, the size of the volume imaged poses a burden, and often new tools that are fully 3D capable and able to work through the big volumes are required.^123^, ^124^ Increasingly, Graphics Processing Units (GPU)^125^ and machine learning tasks are being used to classify pixels and voxels^126^, ^127^ in ‘big data’. However, the advent of Artificial Intelligence (AI) is leading to more complex Deep Learning (DL) tools being employed for mesoscale data.

In the field of AI different implementations of machine learning exist, including DL. For DL, the idea is that an artificial neural network learns to approximate ground truth to carry out analysis tasks.^128^ Different obstacles are in the way of doing this efficiently. On the one hand, training data and ground truth can be challenging to obtain. Often the idea of setting up an AI analysis is to replace tedious and labour‐intensive interpretation of the data by humans. For that purpose, enough data analysed and annotated by humans need to be at hand, and the data need to be similar to the new data that are going to be analysed.^129^ Furthermore, the process of training a deep neural network involves determining the values of the vast number of network parameters using an iterative algorithm and multiple passes over all the training data. This requires considerable computational resources and can take up to days on high‐end machines. However, once the network is trained, analysing similar data is relatively straightforward computationally.

For analysing mesoscale data with DL and other AI strategies, new problems arise. The data sets themselves are rather large, which means that the computationally intensive training task might be exacerbated even more. Smaller subsets of the mesoscale data could be used for training. However, for DL algorithms to work, the samples given to the network for prediction need to come from the same statistical distribution as the samples used during training and therefore need to be representative. As discussed before, inhomogeneities in the data set stemming from the non‐perfect clearing, scattering, and absorption lead to inhomogeneities in the data quality across the sample. Consequently, the size of the training data set will ultimately be a balance between faster training of the model and better generalisation and predictive power of the trained network.

The creation of a DL analysis tool that is very flexible regarding the input and allows reliable detection of features, like (for example) Cellpose, is a great asset.^130^ However, this flexibility comes at the price of very extensive training.

By themselves, the discussed shading and scattering artefacts in mesoscale imaging could be ameliorated using DL strategies. Specifically, for the shading, for example by blood vessels, the artefacts could be detected and the missing information could be filled in by DL approaches. This particular problem is described by image in‐painting, which is the task of realistically completing an image that has missing pixels. Multiple solutions for photographic scenarios have been described.^131^, ^132^, ^133^ We expect that respective solutions for mesoscale imaging will become available soon, as some have already been applied to material microscopy.^134^

Next to absorption and shading, scattering could be tackled computationally,^135^ and interestingly, simulation approaches have been used to guide adaptive optics.^136^ Given the computational difficulty and cost of generating rapid physical corrections, there is also increasing use of AI and DL to correct emission data,^137^ especially in novel hybrid systems^138^ that could also be applied to optical mesoscale imaging.

With multiple groups working on tasks to analyse microscopy and mesoscale data, in the future, strategies like the Model Zoo (https://modelzoo.co/) that contain implementations of already trained networks can help with reproducibility, benchmarking, and finding ready‐made solutions.

In medical imaging, AI finds broad usage ranging from patient organisation^139^ to diagnostics, but two aspects stick out: dosage reduction and diagnostics. For diagnostics, the question boils down to image segmentation, identifying features and classifying objects.^140^ This is in line with microscopy, where AI and related techniques have found broad use for image segmentation.^141^, ^142^, ^143^ Likewise, we expect this to become increasingly relevant for mesoscale imaging.^144^

Additionally, for medical imaging, dosage reduction takes an important role.^145^ OPT can take advantage of these CT strategies from medical imaging, such as compressed sensing and DL approaches, to reconstruct 3D volumes from fewer projection images to reduce the light dose and accelerate 3D image data acquisition. This approach was recently exemplified for in vivo OPT imaging of zebrafish.^146^ Similarly, the use of DL for image restoration, denoising and enhancement of resolution in microscopy applications is promising^136^, ^147^ and can enable faster and more gentle imaging in general and promote live imaging. Related strategies have already been applied to light‐sheet data for isotropic volume imaging.^148^

Although full quantification in medical imaging is ultimately driven by patient benefit, consideration and correction for factors that affect even semi‐quantification of mesoscale microscopy can be considered relevant in the determination of biological structures and functions. Despite their successes, DL and AI are not free from artefacts, and there is a danger that present information is overinterpreted and that the DL networks reproduce only the learned content.

A specific mesoscale challenge for DL and AI approaches could be to take care of specific optical limitations that are difficult to deal with in the real world, like distortions and aberrations.

## 8 CONCLUSIONS

The most important parameter that affects the application of mesoscale imaging techniques is the nature of the sample. By themselves, sample handling, mounting and related issues can be very tricky, and so far, we have seen many studies on optical developments. However, a challenge will be to tackle imaging, and specifically sample handling, to leverage the novel optical approaches for many different biological questions. In the future, we need more robust systems that work for different types of samples and allow us to tackle biological questions in a quantitative manner. In this context, the problem may easily be hidden in detail. Light‐sheet microscopy has been described as well suited for neuroscience imaging.^149^, ^150^ This is mostly because zebrafish brains can be imaged alive and clearing of brain tissue works well. Therefore, neurodevelopment can be imaged without many problems. However, for neurodegenerative diseases, aged animals need to be imaged, and clearing of old brains is more difficult, likely due to the deposition of lipofuscin and other factors. This makes mesoscale imaging much more challenging for Alzheimer's disease research.^24^

Compared to optical imaging, the medical imaging community has been dealing for far longer with the difficulties of extracting quantitative data from images of large samples. Elaborate procedures for estimating tissue attenuation and means for compensation are well established in the medical imaging field, while we are currently missing this in optical imaging to some extent. The creation of specific phantoms and better characterisation of the systems could be a way to tackle this. In addition, direct ways to estimate the attenuation from the sample in place might be required. For that, building on strategies like OPTiSPIM and, for example, combining it with adaptive optics can be a way to develop this further.

Consequently, we need new developments for optical mesoscale imaging, and we already see that developments for the light‐sheet world are recapitulated in mesoscale light‐sheet imaging.^93^, ^151^ The successful approaches of the Mesolens to overcome the century‐old limit of the eyes as the determinant of the field of view demonstrated this impressively. In the future, we can expect that all different permutations of configurations with multiple illuminations and readout arms will also manifest on the mesoscale, and we need to combine them with different readout and analysis strategies to make optical mesoscale imaging more quantitative.

## References

[1] Beghin, A. , Grenci, G. , Rajendiran, H. , Delaire, T. , Raffi, S. B. M. , Blanc, D. , & Viasnoff, V. (2021). High content 3D imaging method for quantitative characterization of organoid development and phenotype. bioRxiv, 2021.03.26.437121.

[2] Dekkers, J. F. , Alieva, M. , Wellens, L. M. , Ariese, H. C. R. , Jamieson, P. R. , Vonk, A. M. , & Rios, A. C. (2019). High‐resolution 3D imaging of fixed and cleared organoids. Nature Protocols, 14(6), 1756–1771. 31053799 10.1038/s41596-019-0160-8

[3] Renner, H. , Grabos, M. , Becker, K. J. , Kagermeier, T. E. , Wu, J. , Otto, M. , & Bruder, J. M. (2020). A fully automated high‐throughput workflow for 3D‐based chemical screening in human midbrain organoids. eLife, 9, e52904. 33138918 10.7554/eLife.52904 PMC7609049

[4] Susaki, E. A. , Shimizu, C. , Kuno, A. , Tainaka, K. , Li, X. , Nishi, K. , & Ueda, H. R. (2020). Versatile whole‐organ/body staining and imaging based on electrolyte‐gel properties of biological tissues. Nature Communications, 11(1), 1982. 10.1038/s41467-020-15906-5 PMC7184626 32341345

[5] Frétaud, M. , Rivière, L. , Job, É. D. , Gay, S. , Lareyre, J.‐J. , Joly, J.‐S. , & Thermes, V. (2017). High‐resolution 3D imaging of whole organ after clearing: Taking a new look at the zebrafish testis. Scientific Reports, 7(1), 43012. 28211501 10.1038/srep43012 PMC5314416

[6] Susaki, E. A. , & Ueda, H. R. (2016). Whole‐body and whole‐organ clearing and imaging techniques with single‐cell resolution: Toward organism‐level systems biology in mammals. Cell Chemical Biology, 23(1), 137–157. 26933741 10.1016/j.chembiol.2015.11.009

[7] Chen, J. , Sivan, U. , Tan, S. L. , Lippo, L. , Angelis, J. D. , Labella, R. , & Kusumbe, A. P. (2021). High‐resolution 3D imaging uncovers organ‐specific vascular control of tissue aging. Science Advances, 7(6), eabd7819. 33536212 10.1126/sciadv.abd7819 PMC7857692

[8] Ertürk, A. (2014). Chapter 5 – High‐resolution 3D imaging of intact transparent organs by 3DISCO. In A. Cornea & P. M. Conn (eds.), Fluorescence microscopy (pp. 65–81). Boston: Academic Press.

[9] Qi, Y. , Yu, T. , Xu, J. , Wan, P. , Ma, Y. , Zhu, J. , & Zhu, D. (2019). FDISCO: Advanced solvent‐based clearing method for imaging whole organs. Science Advances, 5(1), eaau8355. 30746463 10.1126/sciadv.aau8355 PMC6357753

[10] Chen, H. , Huang, T. , Yang, Y. , Yao, X. , Huo, Y. , Wang, Y. , & Guo, Z. V. (2021). Sparse imaging and reconstruction tomography for high‐speed high‐resolution whole‐brain imaging. Cell Reports Methods, 1(6), 100089. 35474896 10.1016/j.crmeth.2021.100089 PMC9017159

[11] Leroy, O. , Leen, E. v. , Girard, P. , Villedieu, A. , Hubert, C. , Bosveld, F. , & Renaud, O. (2021). Multi‐view confocal microscopy enables multiple organ and whole organism live‐imaging. bioRxiv, 2021.05.04.442565. 10.1242/dev.199760 35072204

[12] Olson, P. D. , Zarowiecki, M. , James, K. , Baillie, A. , Bartl, G. , Burchell, P. , & Berriman, M. (2018). Genome‐wide transcriptome profiling and spatial expression analyses identify signals and switches of development in tapeworms. EvoDevo, 9(1), 21. 30455861 10.1186/s13227-018-0110-5 PMC6225667

[13] Dodt, H.‐U. , Leischner, U. , Schierloh, A. , Jährling, N. , Mauch, C. P. , Deininger, K. , & Becker, K. (2007). Ultramicroscopy: Three‐dimensional visualization of neuronal networks in the whole mouse brain. Nature Methods, 4(4), 331–336. 17384643 10.1038/nmeth1036

[14] Affaticati, P. , Le Mével, S. , Jenett, A. , Rivière, L. , Machado, E. , Mughal, B. B. , & Fini, J.‐B. (2018). X‐FaCT: Xenopus‐fast clearing technique. In K. Vleminckx (ed.), Xenopus: Methods and protocols (pp. 233–241). New York, NY: Springer. 10.1007/978-1-4939-8784-9_16 30151770

[15] Kerstens, A. , Corthout, N. , Pavie, B. , Huang, Z. , Vernaillen, F. , Vande Velde, G. , & Munck, S. (2019). A label‐free multicolor optical surface tomography (ALMOST) imaging method for nontransparent 3D samples. BMC Biology, 17(1), 1. 30616566 10.1186/s12915-018-0614-4 PMC6323867

[16] Olivier, N. , Luengo‐Oroz, M. A. , Duloquin, L. , Faure, E. , Savy, T. , Veilleux, I. , & Beaurepaire, E. (2010). Cell lineage reconstruction of early zebrafish embryos using label‐free nonlinear microscopy. Science, 329(5994), 967–971. 20724640 10.1126/science.1189428

[17] Huisken, J. , Swoger, J. , Del Bene, F. , Wittbrodt, J. , & Stelzer, E. H. K. (2004). Optical sectioning deep inside live embryos by selective plane illumination microscopy. Science, 305(5686), 1007–1009. 15310904 10.1126/science.1100035

[18] Tomer, R. , Khairy, K. , Amat, F. , & Keller, P. J. (2012). Quantitative high‐speed imaging of entire developing embryos with simultaneous multiview light‐sheet microscopy. Nature Methods, 9(7), 755–763. 22660741 10.1038/nmeth.2062

[19] Truong, T. V. , Supatto, W. , Koos, D. S. , Choi, J. M. , & Fraser, S. E. (2011). Deep and fast live imaging with two‐photon scanned light‐sheet microscopy. Nature Methods, 8(9), 757–760. 21765409 10.1038/nmeth.1652

[20] Hahn, M. , Nord, C. , Eriksson, M. , Morini, F. , Alanentalo, T. , Korsgren, O. , & Ahlgren, U. (2021). 3D imaging of human organs with micrometer resolution – Applied to the endocrine pancreas. Communications Biology, 4(1), 1–10. 34508173 10.1038/s42003-021-02589-x PMC8433206

[21] Zhao, S. , Todorov, M. I. , Cai, R. , AI ‐Maskari, R. , Steinke, H. , Kemter, E. , & Ertürk, A. (2020). Cellular and molecular probing of intact human organs. Cell, 180(4), 796–812.e19. 32059778 10.1016/j.cell.2020.01.030 PMC7557154

[22] Tyson, A. L. , & Margrie, T. W. (2021). Mesoscale microscopy and image analysis tools for understanding the brain. Progress in Biophysics and Molecular Biology, 168, 81–93. 34216639 10.1016/j.pbiomolbio.2021.06.013 PMC8786668

[23] Guix, F. X. , Sannerud, R. , Berditchevski, F. , Arranz, A. M. , Horré, K. , Snellinx, A. , & De Strooper, B. (2017). Tetraspanin 6: A pivotal protein of the multiple vesicular body determining exosome release and lysosomal degradation of amyloid precursor protein fragments. Molecular Neurodegeneration, 12(1), 25. 28279219 10.1186/s13024-017-0165-0 PMC5345265

[24] Huang, Y. , Skwarek‐Maruszewska, A. , Horré, K. , Vandewyer, E. , Wolfs, L. , Snellinx, A. , & Thathiah, A. (2015). Loss of GPR3 reduces the amyloid plaque burden and improves memory in Alzheimer's disease mouse models. Science Translational Medicine, 7(309), 309ra164. 10.1126/scitranslmed.aab3492 26468326

[25] Li, J. , Czajkowsky, D. M. , Li, X. , & Shao, Z. (2015). Fast immuno‐labeling by electrophoretically driven infiltration for intact tissue imaging. Science Reports, 5(1), 1–7. 10.1038/srep10640 PMC4603706 26013317

[26] Horton, N. G. , Wang, K. , Kobat, D. , Clark, C. G. , Wise, F. W. , Schaffer, C. B. , & Xu, C. (2013). In vivo three‐photon microscopy of subcortical structures within an intact mouse brain. Nature Photonics, 7(3), 205–209. 10.1038/nphoton.2012.336 PMC3864872 24353743

[27] Mahou, P. , Zimmerley, M. , Loulier, K. , Matho, K. S. , Labroille, G. , Morin, X. , & Beaurepaire, E. (2012). Multicolor two‐photon tissue imaging by wavelength mixing. Nature Methods, 9(8), 815–818. 22772730 10.1038/nmeth.2098

[28] Witte, S. , Negrean, A. , Lodder, J. C. , de Kock, C. P. J. , Testa Silva, G. , Mansvelder, H. D. , & Louise Groot, M. (2011). Label‐free live brain imaging and targeted patching with third‐harmonic generation microscopy. PNAS, 108(15), 5970–5975. 21444784 10.1073/pnas.1018743108 PMC3076839

[29] van Rooij, J. , & Kalkman, J. (2019). Large‐scale high‐sensitivity optical diffraction tomography of zebrafish. Biomedical Optics Express, 10(4), 1782–1793. 31086704 10.1364/BOE.10.001782 PMC6484977

[30] Matlock, A. , & Tian, L. (2019). High‐throughput, volumetric quantitative phase imaging with multiplexed intensity diffraction tomography. Biomedical Optics Express, 10(12), 6432–6448. 31853409 10.1364/BOE.10.006432 PMC6913397

[31] Wang, S. , Larina, I. V. , & Larin, K. V. (2020). Label‐free optical imaging in developmental biology [Invited]. Biomed Optics Express, 11(4), 2017–2040. 10.1364/BOE.381359 PMC7173889 32341864

[32] Yang, V. X. D. , Gordon, M. L. , Seng‐Yue, E. , Lo, S. , Qi, B. , Pekar, J. , & Vitkin, I. A. (2003). High speed, wide velocity dynamic range Doppler optical coherence tomography (Part II): Imaging in vivo cardiac dynamics of *Xenopus laevis* . Optics Express, 11(14), 1650–1658. 19466043 10.1364/oe.11.001650

[33] Inoué, S. (2006). Foundations of confocal scanned imaging in light microscopy. In J. B. Pawley (ed.), Handbook of biological confocal microscopy (pp. 1–19). Boston, MA: Springer US.

[34] Legesse, F. B. , Chernavskaia, O. , Heuke, S. , Bocklitz, T. , Meyer, T. , Popp, J. , & Heintzmann, R. (2015). Seamless stitching of tile scan microscope images. Journal of Microscopy, 258(3), 223–232. 25787148 10.1111/jmi.12236

[35] McConnell, G. , Trägårdh, J. , Amor, R. , Dempster, J. , Reid, E. , & Amos, W. B. (2016). A novel optical microscope for imaging large embryos and tissue volumes with sub‐cellular resolution throughout. eLife, 5, e18659. 27661778 10.7554/eLife.18659 PMC5035146

[36] Mcconnell, G. , & Amos, W. B. (2018). Application of the Mesolens for subcellular resolution imaging of intact larval and whole adult Drosophila. Journal of Microscopy, 270(2), 252–258. 29570774 10.1111/jmi.12693 PMC5947746

[37] Rooney, L. M. , Amos, W. B. , Hoskisson, P. A. , & McConnell, G. (2020). Intra‐colony channels in *E. coli* function as a nutrient uptake system. The ISME Journal, 14(10), 2461–2473. 32555430 10.1038/s41396-020-0700-9 PMC7490401

[38] Francis, R. J. , Robb, G. , McCann, L. , Khatri, B. , Keeble, J. , Dagg, B. , & MacLellan‐Gibson, K. (2020). Three‐dimensional in situ morphometrics of *Mycobacterium tuberculosis* infection within lesions by optical mesoscopy and novel acid‐fast staining. Science Reports, 10(1), 1–11. 10.1038/s41598-020-78640-4 PMC7733456 33311596

[39] Schniete, J. , Franssen, A. , Dempster, J. , Bushell, T. J. , Amos, W. B. , & McConnell, G. (2018). Fast optical sectioning for widefield fluorescence mesoscopy with the mesolens based on HiLo microscopy. Science Reports, 8(1), 1–10. 10.1038/s41598-018-34516-2 PMC6215018 30390029

[40] Stelzer, E. H. K. , Strobl, F. , Chang, B.‐J. , Preusser, F. , Preibisch, S. , McDole, K. , & Fiolka, R. (2021). Light sheet fluorescence microscopy. Nature Reviews Methods Primers, 1(1), 1–25.

[41] Keller, P. J. , Schmidt, A. D. , Wittbrodt, J. , & Stelzer, E. H. K. (2008). Reconstruction of zebrafish early embryonic development by scanned light sheet microscopy. Science, 322(5904), 1065–1069. 18845710 10.1126/science.1162493

[42] Keller, P. J. , Schmidt, A. D. , Santella, A. , Khairy, K. , Bao, Z. , Wittbrodt, J. , & Stelzer, E. H. K. (2010). Fast high‐contrast imaging of animal development with scanned light sheet‐based structured illumination microscopy. Nature Methods, 7(8), 637–642. 20601950 10.1038/nmeth.1476 PMC4418465

[43] Huisken, J. , Swoger, J. , Lindek, S. , & Stelzer, E. H. K. (2006). Selective plane illumination microscopy. In J. B. Pawley (ed.), Handbook of biological confocal microscopy (pp. 672–679). Boston, MA: Springer US.

[44] Chen, B.‐C. , Legant, W. R. , Wang, K. , Shao, L. , Milkie, D. E. , Davidson, M. W. , & Betzig, E. (2014). Lattice light‐sheet microscopy: Imaging molecules to embryos at high spatiotemporal resolution. *Science*, 346(6208). 10.1126/science.1257998 PMC4336192 25342811

[45] Dunsby, C. (2008). Optically sectioned imaging by oblique plane microscopy. Optics Express, 16(25), 20306–20316. 19065169 10.1364/oe.16.020306

[46] Bouchard, M. B. , Voleti, V. , Mendes, C. S. , Lacefield, C. , Grueber, W. B. , Mann, R. S. , & Hillman, E. M. C. (2015). Swept confocally‐aligned planar excitation (SCAPE) microscopy for high‐speed volumetric imaging of behaving organisms. Nature Photon, 9(2), 113–119. 10.1038/nphoton.2014.323 PMC4317333 25663846

[47] Hoffmann, M. , & Judkewitz, B. (2019). Diffractive oblique plane microscopy. Optica, 6(9), 1166–1170.

[48] Wang, D. , Wang, D. , Jin, Y. , Jin, Y. , Feng, R. , Feng, R. , & Gao, L. (2019). Tiling light sheet selective plane illumination microscopy using discontinuous light sheets. Optics Express, 27(23), 34472–34483. 31878494 10.1364/OE.27.034472

[49] Chen, Y. , Li, X. , Zhang, D. , Wang, C. , Feng, R. , Li, X. , & Gao, L. (2020). A versatile tiling light sheet microscope for imaging of cleared tissues. *Cell Reports*, 33(5). 10.1016/j.celrep.2020.108349 33147464

[50] Iglesias, I. , & Ripoll, J. (2014). Plenoptic projection fluorescence tomography. Optics Express, 22(19), 23215–23225. 25321790 10.1364/OE.22.023215

[51] Voigt, F. F. , Kirschenbaum, D. , Platonova, E. , Pagès, S. , Campbell, R. A. A. , Kastli, R. , & Helmchen, F. (2019). The mesoSPIM initiative: Open‐source light‐sheet microscopes for imaging cleared tissue. Nature Methods, 16(11), 1105–1108. 31527839 10.1038/s41592-019-0554-0 PMC6824906

[52] Hecht, E. (2002). Optics. Addison‐Wesley, Harlow.

[53] Siedentopf, H. , & Zsigmondy, R. (1903). Über Sichtbarmachung und Grössenbestimmung ultramikroskopischer Teilchen, mit besonderer Anwendung auf Goldrubingläser. Barth.

[54] Lu, R. , Liang, Y. , Meng, G. , Zhou, P. , Svoboda, K. , Paninski, L. , & Ji, N. (2020). Rapid mesoscale volumetric imaging of neural activity with synaptic resolution. Nature Methods, 17(3), 291–294. 32123393 10.1038/s41592-020-0760-9 PMC7192636

[55] Girkin, J. M. , & Carvalho, M. T. (2018). The light‐sheet microscopy revolution. Journal of Optics, 20(5), 053002.

[56] Rawson, S. D. , Maksimcuka, J. , Withers, P. J. , & Cartmell, S. H. (2020). X‐ray computed tomography in life sciences. BMC Biology, 18(1), 21. 32103752 10.1186/s12915-020-0753-2 PMC7045626

[57] Clark, D. P. , & Badea, C. T. (2014). Micro‐CT of rodents: State‐of‐the‐art and future perspectives. Physica Medica, 30(6), 619–634. 24974176 10.1016/j.ejmp.2014.05.011 PMC4138257

[58] Sharpe, J. (2003). Optical projection tomography as a new tool for studying embryo anatomy. Journal of Anatomy, 202(2), 175–181. 12647867 10.1046/j.1469-7580.2003.00155.x PMC1571078

[59] Arranz, A. , Dong, D. , Zhu, S. , Rudin, M. , Tsatsanis, C. , Tian, J. , & Ripoll, J. (2013). Helical optical projection tomography. Optics Express, 21(22), 25912–25925. 24216818 10.1364/OE.21.025912

[60] Chen, L. , McGinty, J. , Taylor, H. B. , Bugeon, L. , Lamb, J. R. , Dallman, M. J. , & French, P. M. W. (2012). Incorporation of an experimentally determined MTF for spatial frequency filtering and deconvolution during optical projection tomography reconstruction. Optics Express, 20(7), 7323–7337. 22453413 10.1364/OE.20.007323

[61] Chen, Y. , Jin, X. , & Xiong, B. (2020). Optical‐aberrations‐corrected light field re‐projection for high‐quality plenoptic imaging. Optics Express, 28(3), 3057–3072. 32121981 10.1364/OE.381720

[62] Ban, S. , Cho, N. H. , Min, E. , Bae, J. K. , Ahn, Y. , Shin, S. , & Jung, W. (2019). Label‐free optical projection tomography for quantitative three‐dimensional anatomy of mouse embryo. Journal of Biophotonics, 12(7), e201800481. 30729697 10.1002/jbio.201800481

[63] Mayer, J. , Robert‐Moreno, A. , Danuser, R. , Stein, J. V. , Sharpe, J. , & Swoger, J. (2014). OPTiSPIM: Integrating optical projection tomography in light sheet microscopy extends specimen characterization to nonfluorescent contrasts. Optics Letters, 39(4), 1053–1056. 24562276 10.1364/OL.39.001053

[64] Karnowski, K. , Ajduk, A. , Wieloch, B. , Tamborski, S. , Krawiec, K. , Wojtkowski, M. , & Szkulmowski, M. (2017). Optical coherence microscopy as a novel, non‐invasive method for the 4D live imaging of early mammalian embryos. Science Reports, 7(1), 1–12. 10.1038/s41598-017-04220-8 PMC5482811 28646146

[65] Ripoll, J. , Koberstein‐Schwarz, B. , & Ntziachristos, V. (2015). Unleashing optics and optoacoustics for developmental biology. Trends in Biotechnology, 33(11), 679–691. 26435161 10.1016/j.tibtech.2015.08.002

[66] Hulst, H. C. , & van de Hulst, H. C. (1981). Light scattering by small particles. Courier Corporation.

[67] Jacques, S. L. (2013). Optical properties of biological tissues: A review. Physics in Medicine and Biology, 58(11), R37–R61. 23666068 10.1088/0031-9155/58/11/R37

[68] Md, Y. H. , & Yamada, Y. (2016). Overview of diffuse optical tomography and its clinical applications. JBO, 21(9), 091312. 27420810 10.1117/1.JBO.21.9.091312

[69] Yan, S. , Yao, R. , Intes, X. , Fang, Q. , & Fang, Q. (2020). Accelerating Monte Carlo modeling of structured‐light‐based diffuse optical imaging via “photon sharing. Optics Letters, 45(10), 2842–2845. 32412482 10.1364/OL.390618 PMC7482422

[70] Cai, Z. , Machado, A. , Chowdhury, R. A. , Spilkin, A. , Vincent, T. , Aydin, Ü. , & Grova, C. (2022). Diffuse optical reconstructions of functional near infrared spectroscopy data using maximum entropy on the mean. Science Reports, 12(1), 2316. 10.1038/s41598-022-06082-1 PMC8831678 35145148

[71] Zhu, C. , & Liu, Q. (2013). Review of Monte Carlo modeling of light transport in tissues. JBO, 18(5), 050902. 10.1117/1.JBO.18.5.050902 23698318

[72] Balasubramaniam, G. M. , Wiesel, B. , Biton, N. , Kumar, R. , Kupferman, J. , & Arnon, S. (2022). Tutorial on the use of deep learning in diffuse optical tomography. Electronics, 11(3), 305.

[73] Branco, S. , Jan, S. , & Almeida, P. (2007). Monte Carlo simulations in small animal PET imaging. Nuclear Instruments and Methods in Physics Research Section A: Accelerators, Spectrometers, Detectors and Associated Equipment, 580(2), 1127–1130. 10.1016/j.nima.2007.06.040 PMC2128617 18836514

[74] Rogers, J. D. , Radosevich, A. J. , Yi, J. , & Backman, V. (2013). Modeling light scattering in tissue as continuous random media using a versatile refractive index correlation function. IEEE Journal of Selected Topics in Quantum Electronics, 20(2), 7000514. 25587211 10.1109/JSTQE.2013.2280999 PMC4289622

[75] Spalteholz, W. (1914). Ueber das Durchsichtigmachen von menschlichen und tierischen Praeparaten und seine theoretischen Bedingungen, S. Hirzel.

[76] Richardson, D. S. , & Lichtman, J. W. (2015). Clarifying Tissue Clearing. Cell, 162(2), 246–257. 26186186 10.1016/j.cell.2015.06.067 PMC4537058

[77] Pende, M. , Vadiwala, K. , Schmidbaur, H. , Stockinger, A. W. , Murawala, P. , Saghafi, S. , & Dodt, H.‐U. (2020). A versatile depigmentation, clearing, and labeling method for exploring nervous system diversity. Science Advances, 6(22), eaba0365. 32523996 10.1126/sciadv.aba0365 PMC7259959

[78] Susaki, E. A. , Tainaka, K. , Perrin, D. , Yukinaga, H. , Kuno, A. , & Ueda, H. R. (2015). Advanced CUBIC protocols for whole‐brain and whole‐body clearing and imaging. Nature Protocols, 10(11), 1709–1727. 26448360 10.1038/nprot.2015.085

[79] Chung, K. , Wallace, J. , Kim, S.‐Y. , Kalyanasundaram, S. , Andalman, A. S. , Davidson, T. J. , & Deisseroth, K. (2013). Structural and molecular interrogation of intact biological systems. Nature, 497(7449), 332–337. 23575631 10.1038/nature12107 PMC4092167

[80] Lee, E. , Choi, J. , Jo, Y. , Kim, J. Y. , Jang, Y. J. , Lee, H. M. , & Sun, W. (2016). ACT‐PRESTO: Rapid and consistent tissue clearing and labeling method for 3‐dimensional (3D) imaging. Science Reports, 6(1), 1–15. 10.1038/srep18631 PMC4707495 26750588

[81] Chen, F. , Tillberg, P. W. , & Boyden, E. S. (2015). Expansion microscopy. Science, 347(6221), 543–548. 25592419 10.1126/science.1260088 PMC4312537

[82] Parra‐Damas, A. , & Saura, C. A. (2020). Tissue clearing and expansion methods for imaging brain pathology in neurodegeneration: From circuits to synapses and beyond. Frontiers in Neuroscience, 14, 914. 33122983 10.3389/fnins.2020.00914 PMC7571329

[83] Costantini, I. , Cicchi, R. , Silvestri, L. , Vanzi, F. , & Pavone, F. S. (2019). In‐vivo and ex‐vivo optical clearing methods for biological tissues: Review. Biomed Optics Express, 10(10), 5251–5267. 10.1364/BOE.10.005251 PMC6788593 31646045

[84] McDole, K. , Guignard, L. , Amat, F. , Berger, A. , Malandain, G. , Royer, L. A. , & Keller, P. J. (2018). In toto imaging and reconstruction of post‐implantation mouse development at the single‐cell level. Cell, 175(3), 859–876.e33. 30318151 10.1016/j.cell.2018.09.031

[85] Tiwari, D. K. , Tiwari, M. , & Jin, T. (2020). Near‐infrared fluorescent protein and bioluminescence‐based probes for high‐resolution in vivo optical imaging. Advanced Materials, 1(5), 967–987.

[86] Veettikazhy, M. , Nylk, J. , Gasparoli, F. , Escobet‐Montalbán, A. , Hansen, A. K. , Marti, D. , & Dholakia, K. (2020). Multi‐photon attenuation‐compensated light‐sheet fluorescence microscopy. *Science Reports*, 10. 10.1038/s41598-020-64891-8 PMC7229186 32415135

[87] Nylk, J. , McCluskey, K. , Preciado, M. A. , Mazilu, M. , Yang, Z. , Gunn‐Moore, F. J. , & Dholakia, K. (2018). Light‐sheet microscopy with attenuation‐compensated propagation‐invariant beams. Science Advances, 4(4), eaar4817. 29740614 10.1126/sciadv.aar4817 PMC5938225

[88] Huisken, J. , & Stainier, D. Y. R. (2007). Even fluorescence excitation by multidirectional selective plane illumination microscopy (mSPIM). Optics Letters, 32(17), 2608–2610. 17767321 10.1364/ol.32.002608

[89] Krzic, U. , Gunther, S. , Saunders, T. E. , Streichan, S. J. , & Hufnagel, L. (2012). Multiview light‐sheet microscope for rapid in toto imaging. Nature Methods, 9(7), 730–733. 22660739 10.1038/nmeth.2064

[90] Taylor, M. A. , Vanwalleghem, G. C. , Favre‐Bulle, I. A. , & Scott, E. K. (2018). Diffuse light‐sheet microscopy for stripe‐free calcium imaging of neural populations. Journal of Biophotonics, 11(12), e201800088. 29920963 10.1002/jbio.201800088

[91] Denk, W. , Strickler, J. H. , & Webb, W. W. (1990). Two‐photon laser scanning fluorescence microscopy. Science, 248(4951), 73–76. 2321027 10.1126/science.2321027

[92] Denk, W. , Piston, D. W. , & Webb, W. W. (2006). Multi‐photon molecular excitation in laser‐scanning microscopy. In J. B. Pawley (ed.), Handbook of biological confocal microscopy (pp. 535–549). Boston, MA: Springer US.

[93] Yu, C.‐H. , Stirman, J. N. , Yu, Y. , Hira, R. , & Smith, S. L. (2020). Diesel2p mesoscope with dual independent scan engines for flexible capture of dynamics in distributed neural circuitry. bioRxiv, 2020.09.20.305508. 10.1038/s41467-021-26736-4 PMC8599518 34789723

[94] Amor, R. , McDonald, A. , Trägårdh, J. , Robb, G. , Wilson, L. , Rahman, N. Z. A. , & McConnell, G. (2016). Widefield two‐photon excitation without scanning: Live cell microscopy with high time resolution and low photo‐bleaching. PLoS One, 11(1), e0147115. 26824845 10.1371/journal.pone.0147115 PMC4732674

[95] Field, J. J. , Carriles, R. , Sheetz, K. E. , Chandler, E. V. , Hoover, E. E. , Tillo, S. E. , & Squier, J. A. (2010). Optimizing the fluorescent yield in two‐photon laser scanning microscopy with dispersion compensation. Optics Express, 18(13), 13661–13672. 20588500 10.1364/OE.18.013661 PMC4151303

[96] Guild, J. B. , Xu, C. , & Webb, W. W. (1997). Measurement of group delay dispersion of high numerical aperture objective lenses using two‐photon excited fluorescence. Applied Optics, 36(1), 397–401. 18250687 10.1364/ao.36.000397

[97] Weninger, W. J. , Geyer, S. H. , Mohun, T. J. , Rasskin‐Gutman, D. , Matsui, T. , Ribeiro, I. , & Müller, G. B. (2006). High‐resolution episcopic microscopy: A rapid technique for high detailed 3D analysis of gene activity in the context of tissue architecture and morphology. Anatomy and Embryology, 211(3), 213–221. 16429276 10.1007/s00429-005-0073-x

[98] Mohun, T. J. , & Weninger, W. J. (2012). Episcopic three‐dimensional imaging of embryos. Cold Spring Harbor Protocols, 2012(6), 641–646. 22661435 10.1101/pdb.top069567

[99] Sharpe, J. , & Wong, R. O. (2011). Imaging in developmental biology: A laboratory manual. Cold Spring Harbor: Cold Spring Harbor Laboratory Press.

[100] Narasimhan, A. , Venkataraju, K. U. , Mizrachi, J. , Albeanu, D. F. , & Osten, P. (2017). Oblique light‐sheet tomography: Fast and high resolution volumetric imaging of mouse brains. bioRxiv. 10.1101/132423

[101] Han, Y. , Kebschull, J. M. , Campbell, R. A. A. , Cowan, D. , Imhof, F. , Zador, A. M. , & Mrsic‐Flogel, T. D. (2018). The logic of single‐cell projections from visual cortex. Nature, 556(7699), 51–56. 29590093 10.1038/nature26159 PMC6585423

[102] Davies, R. , & Kasper, M. (2012). Adaptive optics for astronomy. Annual Review of Astronomy and Astrophysics, 50(1), 305–351.

[103] Pozzi, P. , Gandolfi, D. , Porro, C. A. , Bigiani, A. , & Mapelli, J. (2020). Scattering compensation for deep brain microscopy: The long road to get proper images. *Frontiers in Physics*, 8.

[104] Royer, L. A. , Lemon, W. C. , Chhetri, R. K. , & Keller, P. J. (2018). A practical guide to adaptive light‐sheet microscopy. Nature Protocols, 13(11), 2462–2500. 30367170 10.1038/s41596-018-0043-4

[105] Vinegoni, C. , Razansky, D. , Figueiredo, J.‐L. , Nahrendorf, M. , Ntziachristos, V. , & Weissleder, R. (2009). Normalized Born ratio for fluorescence optical projection tomography. Optics Letters, 34(3), 319–321. 19183644 10.1364/ol.34.000319 PMC2771918

[106] Davis, S. P. X. , Wisniewski, L. , Kumar, S. , Correia, T. , Arridge, S. R. , Frankel, P. , & French, P. M. W. (2018). Slice‐illuminated optical projection tomography. Optics Letters, 43(22), 5555–5558. 30439894 10.1364/OL.43.005555 PMC6238829

[107] Munck, S. , Swoger, J. , Coll‐Lladó, M. , Gritti, N. , & Vande Velde, G. (2021). Maximizing content across scales: Moving multimodal microscopy and mesoscopy toward molecular imaging. Current Opinion in Chemical Biology, 63, 188–199. 34198170 10.1016/j.cbpa.2021.05.003

[108] Shukla, A. K. , & Kumar, U. (2006). Positron emission tomography: An overview. Journal of Medical Physics, 31(1), 13–21. 21206635 10.4103/0971-6203.25665 PMC3003889

[109] Zaidi, H. , Montandon, M.‐L. , & Alavi, A. (2007). Advances in attenuation correction techniques in PET. PET Clinics, 2(2), 191–217. 27157873 10.1016/j.cpet.2007.12.002

[110] Zaidi, H. , & Montandon, M.‐L. (2007). Scatter compensation techniques in PET. PET Clinics, 2(2), 219–234. 27157874 10.1016/j.cpet.2007.10.003

[111] Hutton, B. F. , Buvat, I. , & Beekman, F. J. (2011). Review and current status of SPECT scatter correction. Physics in Medicine and Biology, 56(14), R85–R112. 21701055 10.1088/0031-9155/56/14/R01

[112] McDougald, W. , Vanhove, C. , Lehnert, A. , Lewellen, B. , Wright, J. , Mingarelli, M. , & Tavares, A. A. S. (2019). Standardization of preclinical PET/CT imaging to improve quantitative accuracy, precision and reproducibility: A multi‐center study. Journal of Nuclear Medicine, 61(3), 461–468. 31562220 10.2967/jnumed.119.231308 PMC7067528

[113] Greenwood, H. E. , Nyitrai, Z. , Mocsai, G. , Hobor, S. , & Witney, T. H. (2019). High throughput PET/CT imaging using a multiple mouse imaging system. Journal of Nuclear Medicine, 61(2), 292–297. 31519806 10.2967/jnumed.119.228692 PMC7002164

[114] Sinha, L. , Massanes, F. , Torres, V. C. , Li, C. , Tichauer, K. M. , & Brankov, J. G. (2019). Comparison of time‐ and angular‐domain scatter rejection in mesoscopic optical projection tomography: A simulation study. Biomed Opt Express, 10(2), 747–760. 30800512 10.1364/BOE.10.000747 PMC6377887

[115] Marcos‐Vidal, A. , & Ripoll, J. (2020). Recent advances in optical tomography in low scattering media. Optics and Lasers in Engineering, 135, 106191.

[116] DeWerd, L. A. , & Kissik, M. (eds.) (2014). The phantoms of medical and health physics. New York, NY: Springer.

[117] Del Rosario, M. , Heil, H. S. , Mendes, A. , Saggiomo, V. , & Henriques, R. (2022). The field guide to 3D printing in optical microscopy for life sciences. Advanced Biology, 6, 2100994. 10.1002/adbi.202100994 34693666

[118] Wang, J. , Coburn, J. , Liang, C.‐P. , Woolsey, N. , Ramella‐Roman, J. C. , Chen, Y. , & Pfefer, T. J. (2014). Three‐dimensional printing of tissue phantoms for biophotonic imaging. Optics Letters, 39(10), 3010–3013. 24978260 10.1364/OL.39.003010

[119] Dempsey, L. A. , Persad, M. , Powell, S. , Chitnis, D. , & Hebden, J. C. (2017). Geometrically complex 3D‐printed phantoms for diffuse optical imaging. Biomed Opt Express, 8(3), 1754–1762. 28663863 10.1364/BOE.8.001754 PMC5480578

[120] Wang, K. , Ho, C.‐C. , Zhang, C. , & Wang, B. (2017). A review on the 3D printing of functional structures for medical phantoms and regenerated tissue and organ applications. Engineering, 3(5), 653–662.

[121] Ziemczonok, M. , Kuś, A. , Wasylczyk, P. , & Kujawińska, M. (2019). 3D‐printed biological cell phantom for testing 3D quantitative phase imaging systems. Science Reports, 9(1), 1–9. 10.1038/s41598-019-55330-4 PMC6906528 31827171

[122] Hernandez‐Quintanar, L. , & Rodriguez‐Salvador, M. (2018). Discovering new 3D bioprinting applications: Analyzing the case of optical tissue phantoms. *International Journal of Bioprinting*, 5(1). 10.18063/IJB.v5i1.178 PMC7294689 32596533

[123] Tosi, S. , Bardia, L. , Filgueira, M. J. , Calon, A. , & Colombelli, J. (2020). LOBSTER: An environment to design bioimage analysis workflows for large and complex fluorescence microscopy data. Bioinformatics, 36(8), 2634–2635. 31860062 10.1093/bioinformatics/btz945

[124] Herbert, S. , Valon, L. , Mancini, L. , Dray, N. , Caldarelli, P. , Gros, J. , & Tinevez, J.‐Y. (2021). DProj: A toolbox for local 2D projection and accurate morphometrics of large 3D microscopy images. bioRxiv., 2021.01.15.426809. 10.1186/s12915-021-01037-w PMC8254216 34215263

[125] Haase, R. , Royer, L. A. , Steinbach, P. , Schmidt, D. , Dibrov, A. , Schmidt, U. , & Myers, E. W. (2020). CLIJ: GPU‐accelerated image processing for everyone. Nature Methods, 17(1), 5–6. 31740823 10.1038/s41592-019-0650-1

[126] Mergenthaler, P. , Hariharan, S. , Pemberton, J. M. , Lourenco, C. , Penn, L. Z. , & Andrews, D. W. (2021). Rapid 3D phenotypic analysis of neurons and organoids using data‐driven cell segmentation‐free machine learning. PLOS Computational Biology, 17(2), e1008630. 33617523 10.1371/journal.pcbi.1008630 PMC7932518

[127] Berg, S. , Kutra, D. , Kroeger, T. , Straehle, C. N. , Kausler, B. X. , Haubold, C. , & Kreshuk, A. (2019). ilastik: Interactive machine learning for (bio)image analysis. Nature Methods, 16(12), 1226–1232. 31570887 10.1038/s41592-019-0582-9

[128] Jacquemet, G. (2021). Deep learning to analyse microscopy images. The Biochemist, 43(5), 60–64.

[129] Goodfellow, I. , Bengio, Y. , & Courville, A. (2016). Deep learning. Cambridge, MA: MIT Press.

[130] Stringer, C. , Wang, T. , Michaelos, M. , & Pachitariu, M. (2021). Cellpose: A generalist algorithm for cellular segmentation. Nature Methods, 18(1), 100–106. 33318659 10.1038/s41592-020-01018-x

[131] Pathak, D. , Krähenbühl, P. , Donahue, J. , Darrell, T. , & Efros, A. A. (2016). Context encoders: Feature learning by inpainting. 2016 IEEE Conference on Computer Vision and Pattern Recognition (CVPR) .

[132] Yu, J. , Lin, Z. , Yang, J. , Shen, X. , Lu, X. , & Huang, T. (2019). Free‐form image inpainting with gated convolution. arXiv:1806.03589 [cs].

[133] Yu, J. , Lin, Z. , Yang, J. , Shen, X. , Lu, X. , & Huang, T. S. (2018). Generative image inpainting with contextual attention. arXiv:1801.07892 [cs].

[134] Ma, B. , Ma, B. , Gao, M. , Wang, Z. , Ban, X. , Huang, H. , & Wu, W. (2021). Deep learning‐based automatic inpainting for material microscopic images. Journal of Microscopy, 281(3), 177–189. 32901937 10.1111/jmi.12960

[135] González‐Rodríguez, P. , Kim, A. D. , & Moscoso, M. (2013). Robust depth selectivity in mesoscopic scattering regimes using angle‐resolved measurements. Optics Letters, 38(5), 787–789. 23455299 10.1364/OL.38.000787

[136] Weigert, M. , Subramanian, K. , Bundschuh, S. T. , Myers, E. W. , & Kreysing, M. (2018). Biobeam—Multiplexed wave‐optical simulations of light‐sheet microscopy. PLOS Computational Biology, 14(4), e1006079. 29652879 10.1371/journal.pcbi.1006079 PMC5898703

[137] Xiang, H. , Lim, H. , Fessler, J. A. , & Dewaraja, Y. K. (2020). A deep neural network for fast and accurate scatter estimation in quantitative SPECT/CT under challenging scatter conditions. European Journal of Nuclear Medicine and Molecular Imaging, 47(13), 2956–2967. 32415551 10.1007/s00259-020-04840-9 PMC7666660

[138] Lundervold, A. S. , & Lundervold, A. (2019). An overview of deep learning in medical imaging focusing on MRI. Zeitschrift für Medizinische Physik, 29(2), 102–127. 30553609 10.1016/j.zemedi.2018.11.002

[139] Wagner, J. B. (2019). Artificial Intelligence in Medical Imaging. Radiologic Technology, 90(5), 489–501. 31088949

[140] Oren, O. , Gersh, B. J. , & Bhatt, D. L. (2020). Artificial intelligence in medical imaging: Switching from radiographic pathological data to clinically meaningful endpoints. The Lancet Digital Health, 2(9), e486–e488. 33328116 10.1016/S2589-7500(20)30160-6

[141] Rivenson, Y. , Göröcs, Z. , Günaydin, H. , Zhang, Y. , Wang, H. , & Ozcan, A. (2017). Deep learning microscopy. Optica, 4(11), 1437–1443.

[142] de Haan, K. , Rivenson, Y. , Wu, Y. , & Ozcan, A. (2020). Deep‐learning‐based image reconstruction and enhancement in optical microscopy. Proceedings of the IEEE, 108(1), 30–50.

[143] von Chamier, L. , Laine, R. F. , & Henriques, R. (2019). Artificial intelligence for microscopy: What you should know. Biochemical Society Transactions, 47(4), 1029–1040. 31366471 10.1042/BST20180391

[144] Shaw, M. , Claveau, R. , Manescu, P. , Elmi, M. , Brown, B. J. , Scrimgeour, R. , & Fernandez‐Reyes, D. (2021). Optical mesoscopy, machine learning, and computational microscopy enable high information content diagnostic imaging of blood films. The Journal of Pathology, 255(1), 62–71. 34096621 10.1002/path.5738 PMC12086746

[145] Benjamens, S. , Dhunnoo, P. , & Meskó, B. (2020). The state of artificial intelligence‐based FDA‐approved medical devices and algorithms: An online database. NPJ Digital Medicine, 3(1), 118. 32984550 10.1038/s41746-020-00324-0 PMC7486909

[146] Correia, T. , Lockwood, N. , Kumar, S. , Yin, J. , Ramel, M.‐C. , Andrews, N. , & Arridge, S. (2015). Accelerated optical projection tomography applied to in vivo imaging of zebrafish. PLoS One, 10(8), e0136213. 26308086 10.1371/journal.pone.0136213 PMC4550250

[147] Fang, L. , Monroe, F. , Novak, S. W. , Kirk, L. , Schiavon, C. R. , Yu, S. B. , & Manor, U. (2021). Deep learning‐based point‐scanning super‐resolution imaging. Nature Methods, 1–11. 33686300 10.1038/s41592-021-01080-z PMC8035334

[148] Zhao, F. , Zhu, L. , Fang, C. , Yu, T. , Zhu, D. , & Fei, P. (2020). Deep‐learning super‐resolution light‐sheet add‐on microscopy (Deep‐SLAM) for easy isotropic volumetric imaging of large biological specimens. Biomed Optics Express, 11(12), 7273–7285. 10.1364/BOE.409732 PMC7747920 33408995

[149] Ueda, H. R. , Ertürk, A. , Chung, K. , Gradinaru, V. , Chédotal, A. , Tomancak, P. , & Keller, P. J. (2020). Tissue clearing and its applications in neuroscience. Nature Reviews Neuroscience, 21(2), 61–79. 31896771 10.1038/s41583-019-0250-1 PMC8121164

[150] Ueda, H. R. , Dodt, H.‐U. , Osten, P. , Economo, M. N. , Chandrashekar, J. , & Keller, P. J. (2020). Whole‐brain profiling of cells and circuits in mammals by tissue clearing and light‐sheet microscopy. Neuron, 106(3), 369–387. 32380050 10.1016/j.neuron.2020.03.004 PMC7213014

[151] Orlova, N. , Tsyboulski, D. , Najafi, F. , Seid, S. , Kivikas, S. , Kato, I. , & Saggau, P. (2020). Multiplane Mesoscope reveals distinct cortical interactions following expectation violations. bioRxiv., 2020.10.06.328294.

